# METTL3 modulates colonic epithelium integrity via maintaining the self-renewal and differentiation of Lgr5^+^ stem cell

**DOI:** 10.1093/jmcb/mjae060

**Published:** 2025-01-06

**Authors:** Chenbo Ding, Xinhui Yang, Hua Liu, Manolis Roulis, Huifang Chen, Yunzhu Chen, Hao Xu, Yimeng Gao, Jie Zhong, Hua-Bing Li, Youqiong Ye, Wei Cai, Weiguo Hu, Zhengting Wang

**Affiliations:** Institute of Immunological Innovation & Translation, Chongqing Medical University, Chongqing 400016, China; Shanghai Institute of Immunology, State Key Laboratory of Oncogenes and Related Genes, Shanghai Jiao Tong University School of Medicine, Shanghai 200025, China; Department of Immunobiology, Yale University School of Medicine, New Haven, CT 06520-8055, USA; Department of Gastroenterology, Ruijin Hospital, Shanghai Jiao Tong University School of Medicine, Shanghai 200025, China; Department of Gastroenterology, Ruijin Hospital, Shanghai Jiao Tong University School of Medicine, Shanghai 200025, China; Department of Immunobiology, Yale University School of Medicine, New Haven, CT 06520-8055, USA; Shanghai Institute of Immunology, State Key Laboratory of Oncogenes and Related Genes, Shanghai Jiao Tong University School of Medicine, Shanghai 200025, China; Shanghai Institute of Immunology, State Key Laboratory of Oncogenes and Related Genes, Shanghai Jiao Tong University School of Medicine, Shanghai 200025, China; Department of Immunobiology, Yale University School of Medicine, New Haven, CT 06520-8055, USA; Section of Hematology, Yale Cancer Center and Department of Internal Medicine, Yale University School of Medicine, New Haven, CT 06520-8055, USA; Institute for Regenerative Medicine, Shanghai East Hospital, School of Life Sciences and Technology, Tongji University, Shanghai 200123, China; Department of Gastroenterology, Ruijin Hospital, Shanghai Jiao Tong University School of Medicine, Shanghai 200025, China; Institute of Immunological Innovation & Translation, Chongqing Medical University, Chongqing 400016, China; Shanghai Institute of Immunology, State Key Laboratory of Oncogenes and Related Genes, Shanghai Jiao Tong University School of Medicine, Shanghai 200025, China; Department of Immunobiology, Yale University School of Medicine, New Haven, CT 06520-8055, USA; Shanghai Institute of Immunology, State Key Laboratory of Oncogenes and Related Genes, Shanghai Jiao Tong University School of Medicine, Shanghai 200025, China; Department of General Surgery, Shanghai Minimally Invasive Surgery Center, Shanghai Institute of Immunology, Ruijin Hospital, Shanghai Jiao Tong University School of Medicine, Shanghai 200025, China; Department of General Surgery, Shanghai Minimally Invasive Surgery Center, Shanghai Institute of Immunology, Ruijin Hospital, Shanghai Jiao Tong University School of Medicine, Shanghai 200025, China; Department of Gastroenterology, Ruijin Hospital, Shanghai Jiao Tong University School of Medicine, Shanghai 200025, China

**Keywords:** METTL3, Lgr5^+^ stem cell, intestinal development, inflammation, GRB10, IFRD1

## Abstract

The development and homeostasis of intestinal epithelium are mediated by actively proliferating Lgr5^+^ stem cells, which possess a remarkable self-renewal and differentiation capacity. Recently, our study demonstrated that *N*^6^-methyladenosine (m^6^A) methylation was essential for the survival of colonic stem cells. Here, we show that methyltransferase-like 3 (METTL3) expression is downregulated in the colon mucosa in ulcerative colitis (UC) patients and strongly associated with the differentiation and maturation of goblet cells during inflammation. In mice, depletion of *Mettl3* significantly inhibits the self-renewal and differentiation of Lgr5^+^ stem cells, especially the differentiation and maturation of goblet cells, resulting in intestinal dysplasia and spontaneous inflammation. Mechanistically, *Mettl3* deletion-mediated m^6^A loss facilitates the expression levels of growth factor receptor binding protein 10 (Grb10) and interferon-related developmental regulator 1 (Ifrd1) via increasing their messenger RNA stability. We further demonstrate that the levels of GRB10 and IFRD1 are negatively correlated with METTL3 level in UC samples. Collectively, our data indicate that METTL3 enhances the self-renewal and differentiation of Lgr5^+^ stem cells during intestinal development and inflammation, and thus it may be a potential therapeutic target for UC treatment.

## Introduction

Homeostasis of the gastrointestinal tract is maintained by continuous replacement from epithelial stem cells and subsequent high organization and rapid migration (Vermeulen and  Snippert, 2014). Lgr5^+^ stem cells are known to reside at the crypt bottom and produce the rapidly proliferating transit amplifying (TA) cells, which differentiate into different intestinal epithelial cell (IEC) subsets (Barker et al., 2007, 2008; Barker, 2014). The Wnt and Notch signaling pathways function together to maintain stem cells, control proliferation, and govern cell fate decisions (Crosnier et al., 2006; Hong et al., 2016). In addition, signalling by several growth factors is associated with intestinal growth and development, such as epidermal growth factor (EGF), transforming growth factor, and platelet-derived growth factor (Sancho et al., 2004; Crosnier et al., 2006; Noah et al., 2011). In the structural organization of small intestine, Lgr5^+^ stem cells are intercalated with paneth cells at the crypt base (Barker, 2014). Paneth cells could maintain and support the self-renewal and differentiation of Lgr5^+^ stem cells during homeostasis of small intestine, while upon tissue injury, paneth cells switch to a proliferating state, suggesting that small intestine has strong plasticity (Sato et al., 2011; Roth et al., 2012; Xu et al., 2022). In contrast, colonic epithelium is characterized by a high density of goblet cells and the absence of paneth cells (Barker, 2014). Goblet cells are critical for the maintenance of the colonic barrier through the production of mucus and anti-microbial peptides. Although it has been known that the self-renewal and differentiation of Lgr5^+^ stem cells are the foundation to regulate intestinal development, homeostasis, and tissue injury such as inflammatory bowel diseases (IBD), the underlying molecular mechanism is largely unknown.

Accumulating evidence has supported the idea that abnormal epigenetic changes greatly contribute to the development of IBD (Nakayama et al., 2022; Xu et al., 2022), though mechanistically poorly explored. *N*^6^-methyladenosine (m^6^A) is the most prevalent internal modification in eukaryotic messenger RNAs (mRNAs) (Wei et al., 1975). m^6^A RNA methylation is dynamically catalyzed by a multicomponent methyltransferase complex, such as methyltransferase-like 3 (METTL3), methyltransferase-like 14 (METTL14), and Wilms’ tumor 1-associating protein (WTAP), and a demethyltransferase complex such as fat mass- and obesity-associated protein (FTO) and alkylation repair homolog protein 5 (ALKBH5) (Fu et al., 2014). Although recent studies have shown the critical roles of m^6^A in the survival and stemness of intestinal stem cells (ISCs) during intestinal development and tumorigenesis (Han et al., 2020; Chen et al., 2021; Zhang et al., 2022), how m^6^A mRNA modification controls the self-renewal and differentiation of Lgr5^+^ stem cells and then affects intestinal development, homeostasis, and IBD are still unclear.

In this study, we demonstrated that METTL3 is downregulated in IBD patients and strongly associated with the development and differentiation of goblet cells. The depletion of *Mettl3* dramatically restricts intestinal development and goblet cell differentiation and induces spontaneous colitis in mice. Mechanistically, METTL3-mediated m^6^A modification mainly suppresses the mRNA stabilization of growth factor receptor binding protein 10 (GRB10) and interferon-related developmental regulator 1 (IFRD1), which are involved in cell growth and differentiation.

## Results

### METTL3 is downregulated in the colon mucosa of ulcerative colitis and associated with the development of goblet cell

**Figure 1:**
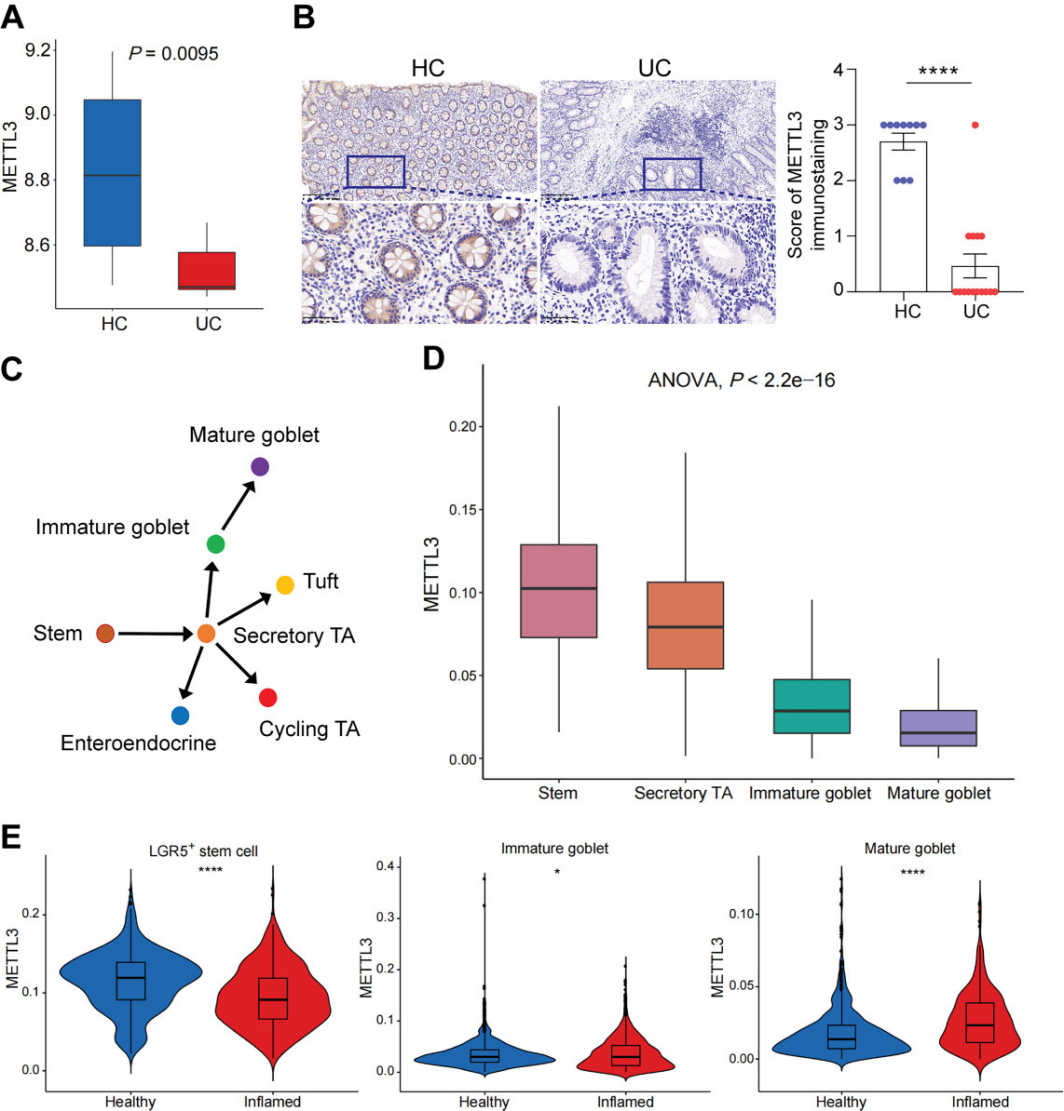
METTL3 is downregulated in the colon mucosa of UC and associated with the development of goblet cell. (**A**) METTL3 expression in colon mucosa of UC patients and healthy controls (HC) based on bulk RNA-seq database (GSE179128). (**B**) Representative images and statistical analysis of IHC staining for METTL3 in colon mucosa of UC patients and HC. Scale bar, 200 μm (upper) and 50 μm (bottom). (**C**) Intestinal epithelial differentiation. Shown is the inferred differentiation trajectory for secretory lineages. (**D**) METTL3 expression in intestinal secretory lineages based on scRNA-seq database (SCP259). (**E**) METTL3 expression in LGR5^+^ stem cells, immature goblet cells, and mature goblet cells from health individuals and inflamed patients based on scRNA-seq database (SCP259). Data are presented as mean ± SEM (**B**) or mean ± SD (**A, D**, and **E**). Wald test (**A**), two-tailed unpaired Student's *t*-test (**B** and **E**), or one-way ANOVA (**D**); *P < 0.05; ****P < 0.0001.

To investigate the role of m^6^A RNA modification in IBD development, we analyzed its methyltransferase and demethyltransferase complexes in IBD patients based on database (GSE179128). Among the m^6^A binding proteins, including METTL3, METTL14, WTAP, FTO, and ALKBH5, the expression of METTL3 was most significantly downregulated in IBD patients (Supplementary Figure S1A). Furthermore, only METTL3 was downregulated in stem cells in inflamed colon tissues compared with healthy colons (SCP259) (Supplementary Figure S1B), suggesting that METTL3 might play a specific role in the function of stem cells during intestinal inflammation. To explore the potential role of METTL3 in the pathogenesis of IBD, we further analyzed its expression in intestinal mucosa from patients with ulcerative colitis (UC) and healthy controls based on database (GSE179128). METTL3 expression was markedly decreased in inflamed colon mucosa from patients with active UC compared with uninflamed mucosa from healthy controls (Figure 1A; Supplementary Figure S2A). Subsequently, we confirmed that METTL3 expression was lower in inflamed colon mucosa from UC patients than in uninflamed mucosa from health individuals by immunohistochemical (IHC) staining (Figure 1B).

Core mucus structural components are reduced in active UC, which is associated with the decreased number of sentinel goblet cells and attenuation of the goblet cell secretory response to microbial challenge (Van Der Post et al., 2019). Secretory epithelial lineages, here specially for goblet cells, are differentiated from secretory TA cells, which are derived from Lgr5^+^ stem cells (Smillie et al., 2019; Figure 1C). It has been known that mature goblet cells and goblet progenitors (secretory TA) are reduced during inflammation (Supplementary Figure S2B; Smillie et al., 2019; Van Der Post et al., 2019). Then, we analyzed METTL3 expression in IECs based on single-cell RNA sequencing (scRNA-seq) database (SCP259) and found that METTL3 was mainly expressed in stem cells and TA cells (Supplementary Figure S2C). To explore whether METTL3 has the potential role in the development and differentiation of goblet cells, we further investigated the dynamic changes of METTL3 expression in secretory epithelial lineages associated with the development of goblet cells. Interestingly, METTL3 expression decreased gradually with the differentiation and maturation of goblet cells, implying an important function of METTL3 in the development and homeostasis of IECs (Figure 1D). Consistently, METTL3 expression levels significantly changed in stem cells, immature goblet cells, and mature goblet cells in IBD samples compared to healthy controls (Figure 1E; Supplementary Figure S2D). Collectively, these data suggest that intestinal epithelial METTL3 may play an important role in the pathogenesis of IBD and the development and differentiation of goblet cells.

### Mettl3 depletion in IECs induces poor intestinal development and differentiation and spontaneous inflammation

**Figure 2:**
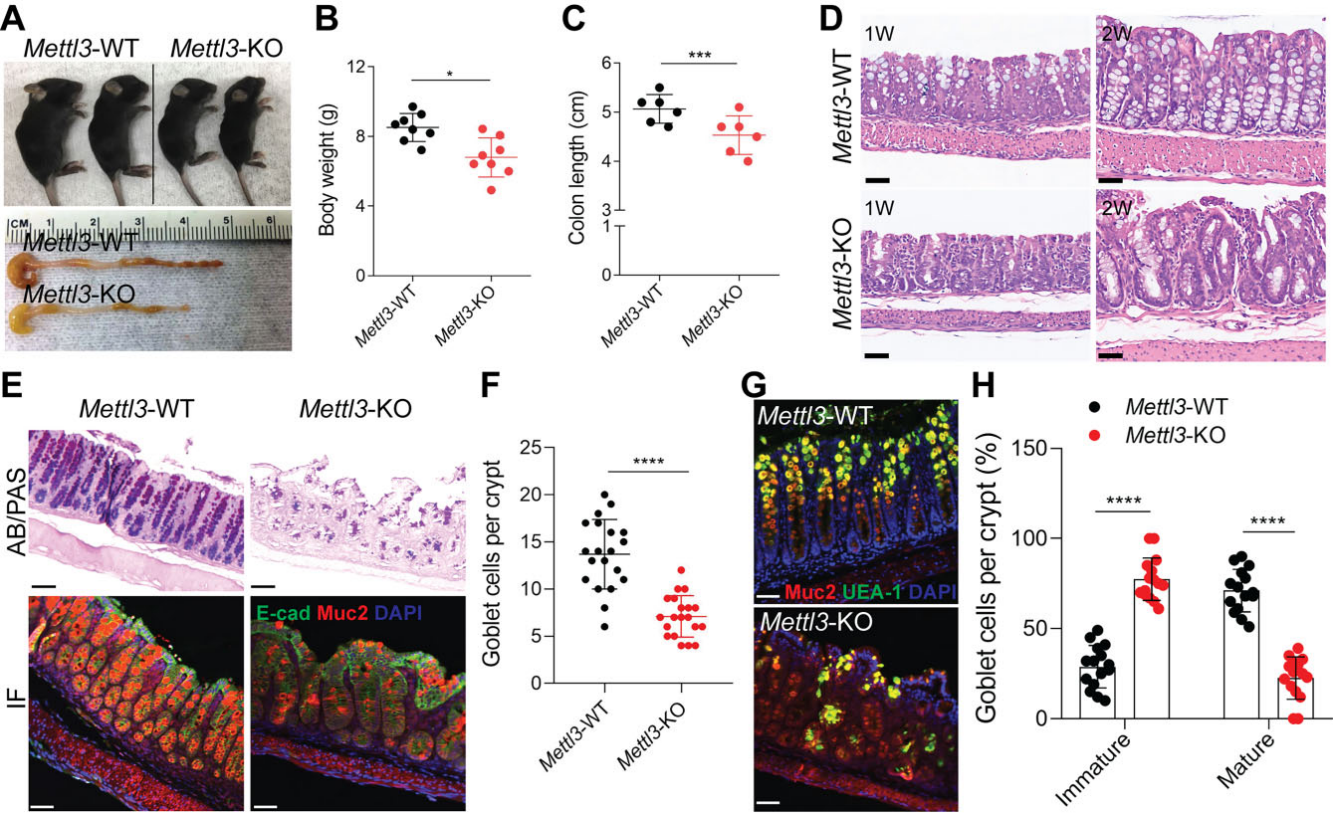
*Mettl3* depletion in IECs induces poor intestinal development and differentiation. (**A**) Representative images of body size (upper) and colon length (bottom) from 2-week-old *Mettl3*-WT and *Mettl3*-KO mice. (**B**) Statistical analysis of body weight in 2-week-old *Mettl3*-WT (*n* = 8) and *Mettl3*-KO (*n* = 8) mice. (**C**) Statistical analysis of colon length in 2-week-old *Mettl3*-WT (*n* = 6) and *Mettl3*-KO (*n* = 6) mice. (**D**) Representative H&E staining images of distal colon sections from 1- and 2-week-old *Mettl3*-WT and *Mettl3*-KO mice. Scale bar, 50 μm. (**E**) Representative AB/PAS and immunofluorescence (IF) staining images of distal colon sections obtained from 2-week-old *Mettl3*-WT (*n* = 5) and *Mettl3*-KO (*n* = 5) mice. Note the reduction in PAS^+^ and Muc2^+^ mature goblet cells in *Mettl3*-KO mice. Scale bar, 50 μm. (**F**) Statistical analysis of goblet cell numbers in **E**. (**G**) Representative confocal images of immunostaining for UEA-1 and Muc2 of distal colon sections from 2-week-old *Mettl3*-WT (*n* = 5) and *Mettl3*-KO (*n* = 5) mice. Scale bar, 50 μm. (**H**) Percentages of immature and mature goblet cells in distal colon sections stained as in **G**. Immature goblet cells were scored as UEA-1^low/−^ cells containing low area (<10 μm in diameter) of Muc2 staining, and mature goblet cells were scored as cells containing large area (>10 μm in diameter) of Muc2 and UEA-1 staining. Data are presented as mean ± SD. Two-tailed unpaired Student's *t*-test; *P < 0.05; ***P < 0.001; ****P < 0.0001.

To delineate the role of METTL3 in intestinal development and differentiation, we conditionally deleted *Mettl3* in IECs by generating *Villin-Cre^+^;Mettl3^f/f^* mice (Supplementary Figure S3A–C). Remarkably, *Villin-Cre*^+^;*Mettl3^f/f^* (hereafter called *Mettl3*-KO) mice had significantly lower body weight compared with *Mettl3^f/f^* (hereafter called *Mettl3*-WT) littermates at 2 weeks old (Figure 2A and B). Additionally, *Mettl3*-KO mice showed a marked decrease in colon length compared with *Mettl3*-WT littermates (Figure 2A and C). To evaluate impacts of *Mettl3* depletion on the intestinal morphology and histology, hematoxylin–eosin (H&E) staining was performed. Surprisingly, compared with *Mettl3*-WT mice, colon dysplasia and loss of goblet cells were found in *Mettl3*-KO mice (Figure 2D). Alcian blue/periodic acid schiff (AB/PAS) and immunofluorescence staining further confirmed that *Mettl3* deletion significantly reduced the number and size of goblet cells in colon (Figure 2E and F). To explore whether *Mettl3* controls the maturation of goblet cells, immunofluorescence staining was performed to assess fucosylated glycoproteins with the lectin Ulex europaeus agglutinin-1 (UEA-1) and the abundant goblet cell mucin Muc2, both of which accumulate in mature goblet cells (Nowarski et al., 2015). *Mettl3*-KO mice exhibited an increase of immature goblet cells, determined by small area of Muc2 staining (<10 mm in diameter) in UEA-1^low/–^ cells and decrease in large mature Muc2^+^UEA-1^+^ goblet cells, compared to *Mettl3*-WT littermates (Figure 2G and H). Furthermore, we analyzed the publicly available scRNA-seq in IECs of *Mettl3*-KO (GSE186913) and found a markedly reduced percentage of mature goblet cells in *Mettl3*-KO IECs compared to *Mettl3*-WT IECs (Supplementary Figure S3D), confirming that *Mettl3* controls the maturation of goblet cells.

Goblet cells are critical for the maintenance of the colonic barrier and the inhibition of inflammation. To explore whether loss of *Mettl3* in IECs drives intestinal dyshomeostasis and inflammation, we performed intestinal permeability assay and found that *Mettl3*-KO mice had the impaired intestinal epithelial barrier (Supplementary Figure S3E). In addition, severe infiltration of inflammatory cells, crypt architectural distortion, and epithelial ulceration were observed in the colon of adult *Mettl3*-KO mice (Supplementary Figure S4A). Although no obvious infiltration of inflammatory cells was found in the small intestine, *Mettl3*-KO mice displayed hyperplasia of Peyer's patches (Supplementary Figure S4B), which is an important indicator of small intestinal inflammation (Elinav et al., 2011). Furthermore, we analyzed mucosal cytokines and immune cell infiltration. Of note, *Mettl3*-KO mice had more immune cell infiltration and higher inflammatory cytokine production compared with *Mettl3*-WT littermates (Supplementary Figure S4C and D). These results strongly support that *Mettl3* deletion in IECs impairs intestinal development and differentiation, which provides favorable conditions for inflammation and induces colitis.

### Mettl3 deletion suppresses the self-renewal and differentiation of ISCs

**Figure 3:**
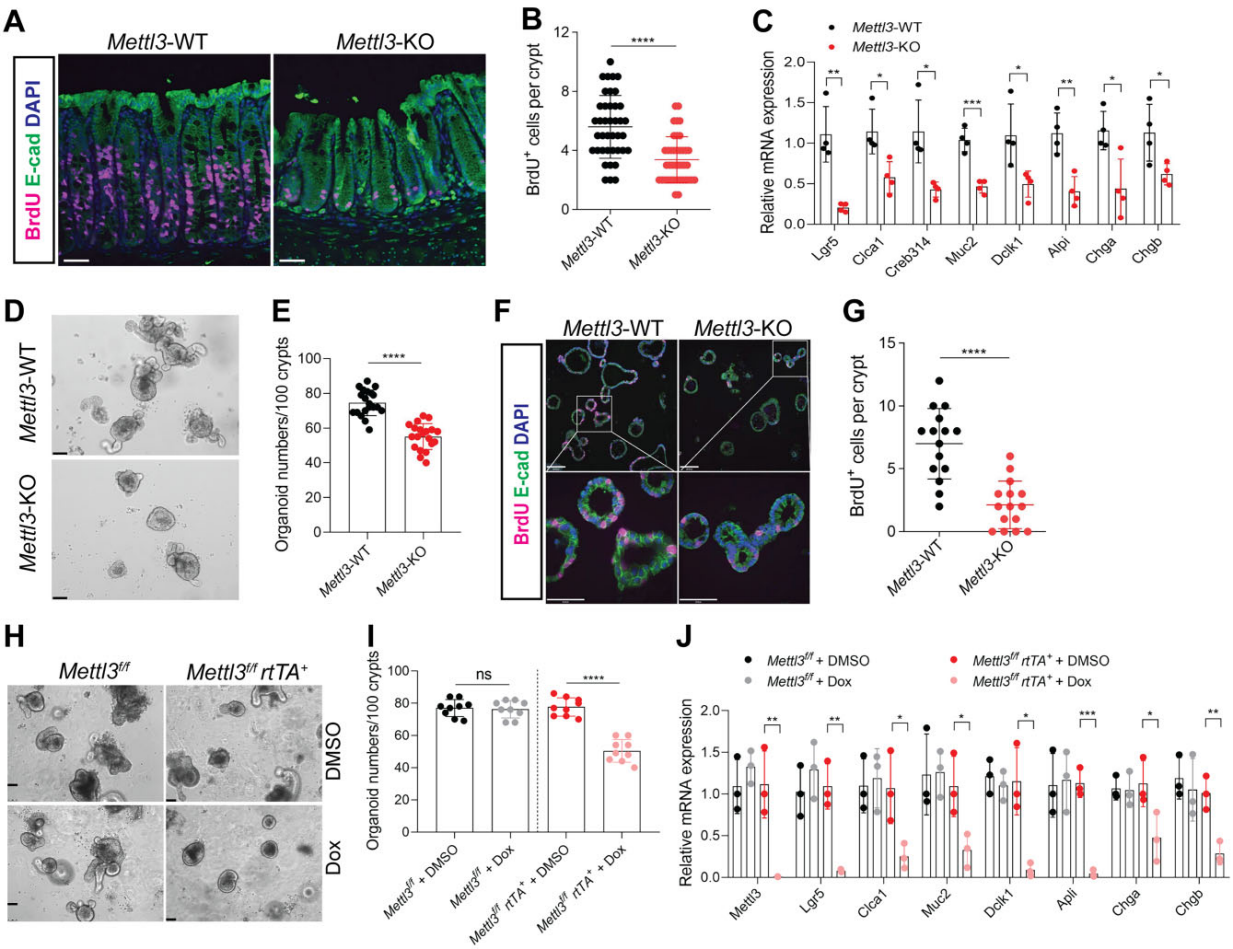
Loss of *Mettl3* suppresses the growth and self-renewal of ISCs. (**A** and **B**) Representative images and quantification of colon crypt proliferation, marked by BrdU incorporation in 2-week-old *Mettl3*-WT (*n* = 5) and *Mettl3*-KO (*n* = 5) mice. Scale bar, 50 μm. (**C**) qPCR analysis of mRNA levels of IEC maker genes including *Lgr5, Clca1, Creb314, Muc2, Dclk1, Alpi, Chga*, and *Chgb* in IECs isolated from 2-week-old *Mettl3*-WT (*n* = 4) and *Mettl3*-KO (*n* = 4) mice. (**D** and **E**) Representative images and quantification of colonic organoids derived from 2-week-old *Mettl3*-WT (*n* = 3) and *Mettl3*-KO (*n* = 3) mice on Day 7 of culture. Scale bar, 100 μm. (**F** and **G**) Representative images and quantification of crypt proliferation of colonic organoids labelled by BrdU, derived from 2-week-old *Mettl3*-WT (*n* = 3) and *Mettl3*-KO (*n* = 3) mice. Scale bar, 100 μm (upper) and 50 μm (bottom). (**H** and **I**) Representative images and quantification of colonic organoids derived from *Mettl3^f/f^* (*n* = 3) and *Mettl3^f/f^ rtTA^+^* (*n* = 3) mice treatment with either DMSO or Dox. Scale bar, 75 μm. (**J**) qPCR analysis of *Mettl3, Lgr5, Clca1, Creb314, Muc2, Dclk1, Alpi, Chga*, and *Chgb* mRNA levels in colonic organoids derived from *Mettl3^f/f^* (*n* = 3) and *Mettl3^f/f^ rtTA^+^* (*n* = 3) mice treatment with either DMSO or Dox. Data are presented as mean ± SD. Two-tailed unpaired Student's *t*-test; *P < 0.05; **P < 0.01; ***P < 0.001; ****P < 0.0001; ns, no significance.

Considered the strong plasticity and inconspicuous phenotype of small intestine after *Mettl3* depletion, we then focused on the colon in the following study. Lgr5^+^ stem cells are the key foundation for the development and homeostasis of the intestine, which differentiate into different IEC subsets via producing TA cells (Barker et al., 2007, 2008; Barker, 2014). To explore whether poor colon development and differentiation is due to the impaired function of stem cells in *Mettl3*-KO mice, we first labeled the IECs *in vivo* with BrdU, a thymidine analogue used to identify proliferating cells. As shown in Figure 3A and B, the number of BrdU^+^ cells was markedly lower in the *Mettl3*-KO group than in the *Mettl3*-WT group. Then, quantitative real-time polymerase chain reaction (qPCR) results demonstrated that *Mettl3* depletion in IECs significantly reduced the mRNA levels of specific markers of colonic epithelial cell subsets (Figure 3C). Moreover, loss of *Mettl3* restricted the growth and self-renewal of stem cell considerably, revealed by the significantly decreased colonic organoid growth and BrdU^+^ cell number in organoid crypts (Figure 3D–G; Supplementary Figure S5). Whole crypts isolated from *Mettl3*-KO mice exhibited distinguishable difference in epithelial composition compared with the *Mettl3*-WT controls *in vivo*, which might affect the results of organoid culture. Hence, we generated *Mettl3^f/f^ rtTA-Cre* mouse strain (hereafter called *Mettl3^f/f^ rtTA^+^*), which enables conditional deletion of *Mettl3* under the treatment of doxycycline (Dox). Both the colonic organoid growth and stem cell differentiation marker gene expression were obviously reduced in the Dox-treated *Mettl3^f/f^ rtTA^+^*group compared with controls (Figure 3H–J). These results indicate that *Mettl3* depletion inhibits the self-renewal and differentiation of ISCs.

### Loss of Mettl3 in Lgr5^+^ stem cell inhibits its self-renewal and differentiation and promotes dextran sulfate sodium salt-induced colitis

**Figure 4:**
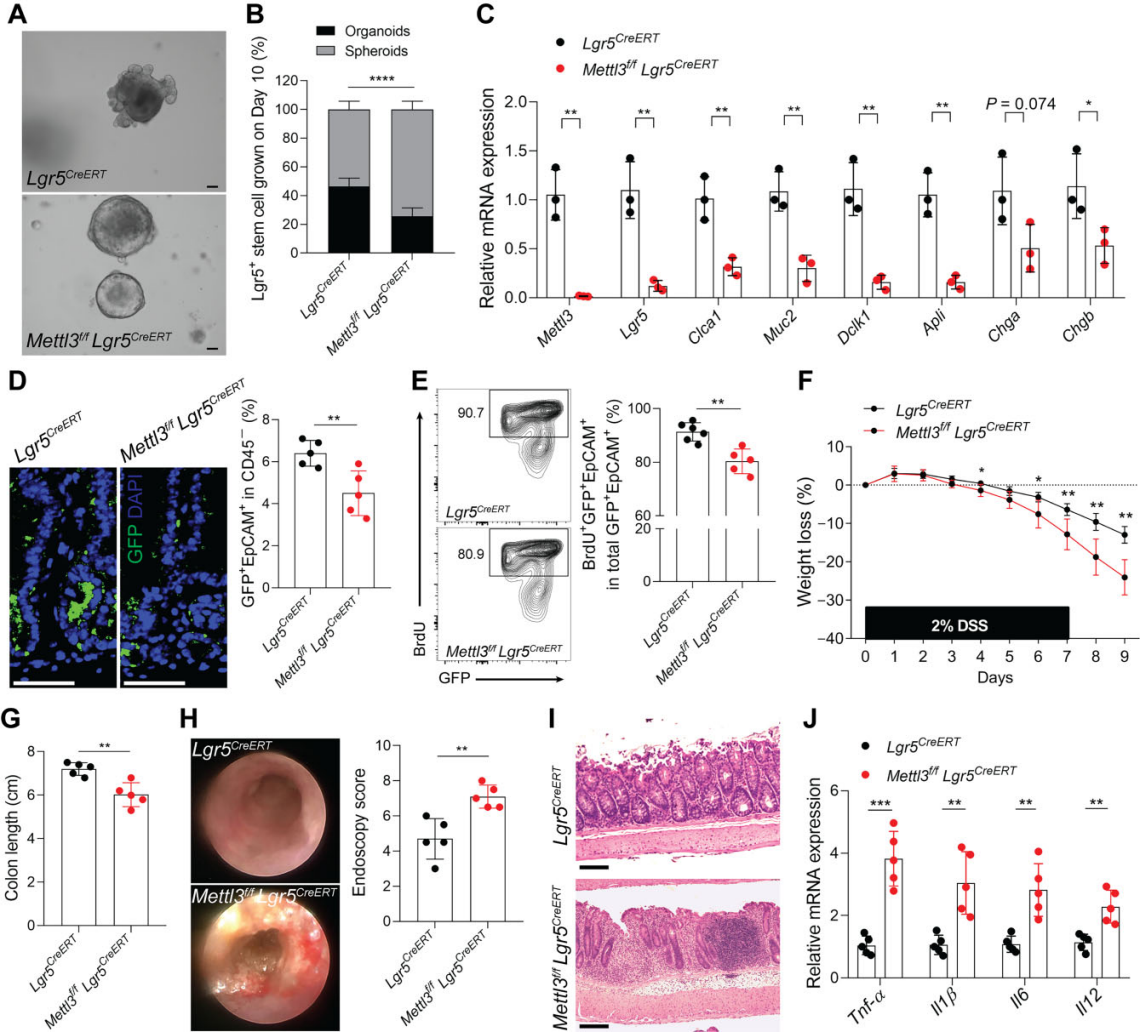
*Mettl3* depletion in Lgr5^+^ cell inhibits its self-renewal and differentiation and promotes DSS-induced colitis. (**A** and **B**) Representative images and quantification of Lgr5^+^ single cell organoids derived from either *Lgr5^CreERT^*(*n* = 3) or *Mettl3^f/f^ Lgr5^CreERT^*(*n* = 3) mice on Day 10 of culture. Scale bar, 100 μm. (**C**) qPCR analysis of *Mettl3, Lgr5, Clca1, Creb314, Muc2, Dclk1, Alpi, Chga*, and *Chgb* mRNA levels in organoids derived from *Lgr5^CreERT^*(*n* = 3) and *Mettl3^f/f^ Lgr5^CreERT^*(*n* = 3) mice. (**D**) Representative images of GFP^+^ Lgr5^+^ stem cells (left) and the percentage of GFP^+^EpCAM^+^ Lgr5^+^ stem cells (right) in live CD45-negative IECs from *Lgr5^CreERT^*(*n* = 5) and *Mettl3^f/f^ Lgr5^CreERT^*(*n* = 5) mice treated with tamoxifen daily for 7 days and rested for 7 days. Scale bar, 30 μm. (**E**) Representative flow cytometry data (left) and the percentage of BrdU^+^GFP^+^EpCAM^+^ Lgr5^+^ stem cells (right) in total live Lgr5^+^ stem cells from *Lgr5^CreERT^*(*n* = 5) and *Mettl3^f/f^ Lgr5^CreERT^*(*n* = 6) mice treated with tamoxifen daily for 7 days and rested for 7 days. (**F**) Weight loss of *Lgr5^CreERT^* (*n* = 5) and *Mettl3^f/f^ Lgr5^CreERT^*(*n* = 5) mice treated with 2% DSS for 7 days. Note: before the course of 7-day 2% DSS administration, *Lgr5^CreERT^* and *Mettl3^f/f^ Lgr5^CreERT^*mice were treated with tamoxifen daily for 7 days and rested for 7 days. (**G**) Gross pathology of colons on Day 9 post-DSS in *Lgr5^CreERT^* (*n* = 5) and *Mettl3^f/f^ Lgr5^CreERT^*(*n* = 5) mice. (**H**) Representative endoscopic view (left) and colonoscopy severity score (right) of *Lgr5^CreERT^*(*n* = 5) and *Mettl3^f/f^ Lgr5^CreERT^*(*n* = 5) littermates on Day 7 post-DSS. (**I**) Representative H&E staining images of colon sections obtained from co-housed *Lgr5^CreERT^*and *Mettl3^f/f^ Lgr5^CreERT^* littermates given DSS and assessed on Day 9. Scale bar, 50 μm. (**J**) qPCR analysis of pro-inflammatory-related genes in *Lgr5^CreERT^*(*n* = 5) and *Mettl3^f/f^ Lgr5^CreERT^*(*n* = 5) mice given DSS and assessed on Day 9. Data are presented as mean ± SD. Two-tailed unpaired Student's *t*-test (**B, C, D, E, G, H**, and **J**) or two-tailed Mann–Whitney *U* test (**F**); *P < 0.05; **P < 0.01; ****P < 0.0001.

Lgr5^+^ cells constitute multipotent stem cells that generate all cell types of the epithelium (Barker et al., 2007). To investigate the role of *Mettl3* in Lgr5^+^ stem cells, we generated *Mettl3^f/f^;Lgr5-eGFP-IRES-Cre^ERT2^* mice (hereafter called *Mettl3^f/f^ Lgr5^CreERT^*). We first determined whether *Mettl3* deficiency in Lgr5^+^ stem cells controls the growth, self-renewal, and differentiation. The Lgr5^+^ single cell organoids displayed a significantly decreased growth rate and hardly formed crypt in the absence of *Mettl3* (Figure 4A and B). Moreover, marker gene expression levels of colonic epithelial cell subsets were markedly reduced in organoids derived from *Mettl3^f/f^ Lgr5^CreERT^*mice (Figure 4C). It has been well-documented that rapidly proliferative and regenerative ISCs ensure intestinal epithelial homeostasis during injury repair. However, complete depletion of Lgr5^+^ stem cells failed to produce a long-term impact on crypt homeostasis due to the existence of an alternative stem cell pool (Tian et al., 2011). To examine whether loss of *Mettl3* suppresses the self-renewal of Lgr5^+^ ISCs, we counted the number of Lgr5^+^ cells and analyzed the percentage of BrdU^+^Lgr5^+^ cells in *Mettl3^f/f^ Lgr5^CreERT^*and *Lgr5^CreERT^*mice via flow cytometry analysis. We found a significantly reduction in the number and proliferation capability of Lgr5^+^ cells in *Mettl3^f/f^ Lgr5^CreERT^* mice compared to *Lgr5^CreERT^*mice (Figure 4D and E). To gain a better functional insight into the effect of *Mettl3* depletion on Lgr5^+^ stem cells, we treated the mice with dextran sulfate sodium salt (DSS) to accelerate the deterioration and dyshomeostasis of IECs. *Mettl3^f/f^ Lgr5^CreERT^* mice developed more severe colitis than *Lgr5^CreERT^* mice, judged by weight loss (Figure 4F), colon length (Figure 4G), endoscopy score (Figure 4H), inflammatory cell infiltration (Figure 4I), and mRNA levels of inflammation-related factors (Figure 4J). Collectively, these data suggest that *Mettl3* depletion in Lgr5^+^ stem cells inhibits the self-renewal and differentiation and promotes DSS-induced colitis.

### Mettl3 deficiency upregulates the expression of Grb10 and Ifrd1

**Figure 5:**
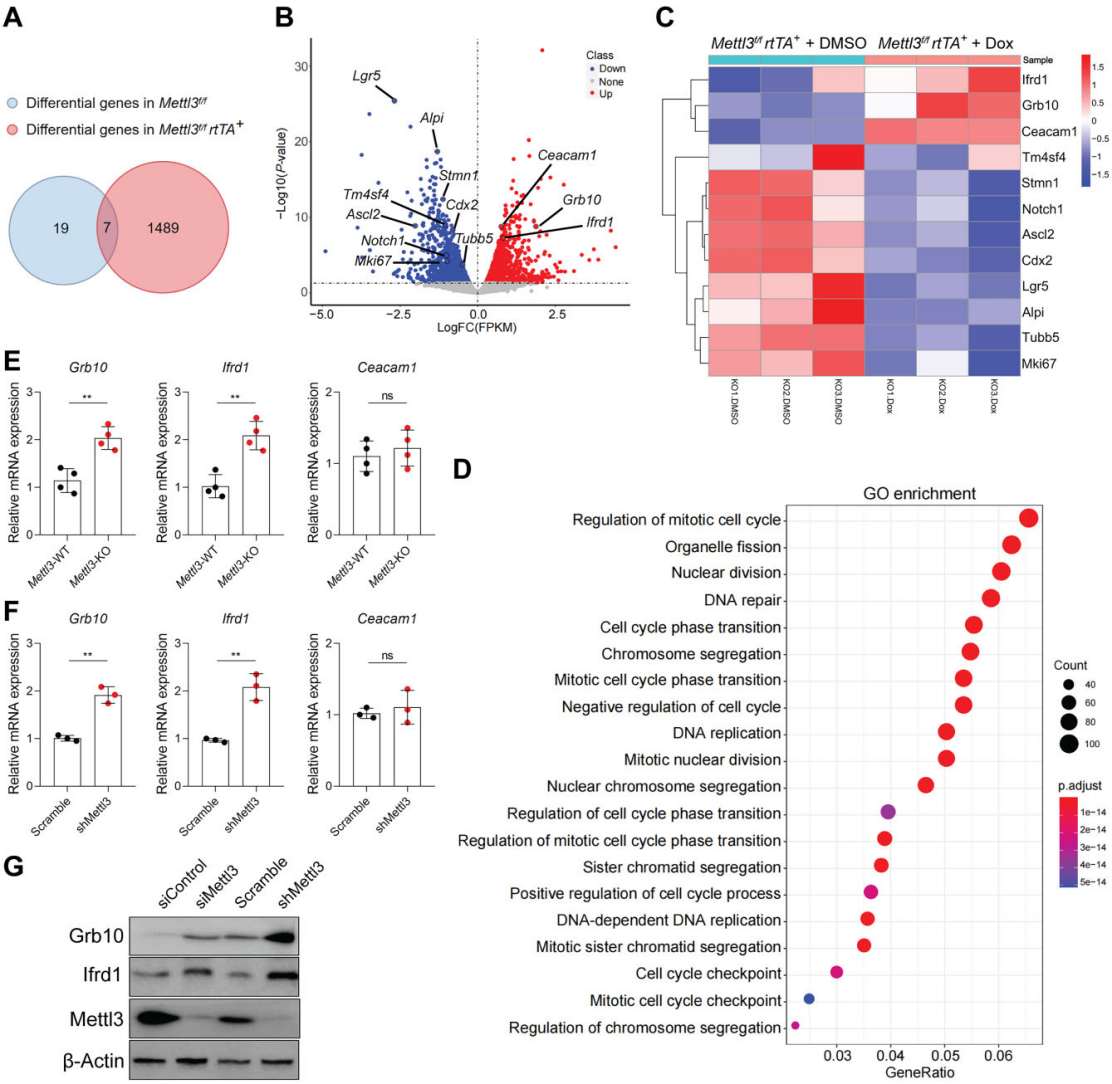
*Mettl3* deficiency upregulates the expression of Grb10 and Ifrd1. (**A**) Venn diagram showing the overlap of DEGs between DMSO- and Dox-treated colonic organoids derived from *Mettl3^f/f^* and *Mettl3^f/f^ rtTA^+^* mice based on RNA-seq data. (**B**) Volcano plot showing RNA-seq data of DMSO- and Dox-treated colonic organoids derived from *Mettl3^f/f^ rtTA^+^* mice. Downregulated genes, such as *Lgr5, Ascl2, Stmn1, Tubb5, Cdx2, Notch1, Tm4sf4, Alpi*, and *Mki67*, and top upregulated genes *Grb10, Ifrd1*, and *Ceacam1* containing high m^6^A peaks are highlighted. (**C**) Heatmap showing *Lgr5, Ascl2, Stmn1, Tubb5, Cdx2, Notch1, Tm4sf4, Alpi*, and *Mki67, Grb10, Ifrd1*, and *Ceacam1* gene expression levels in DMSO- and Dox-treated colonic organoids derived from *Mettl3^f/f^ rtTA^+^* mice. (**D**) GO enrichment analysis of downregulated genes in Dox-treated colonic organoids derived from *Mettl3^f/f^ rtTA^+^* mice. (**E**) qPCR analysis of *Grb10, Ifrd1*, and *Ceacam1* mRNA levels in IECs isolated from 2-week-old *Mettl3*-WT (*n* = 4) and *Mettl3*-KO (*n* = 4) mice. (**F**) qPCR analysis of *Grb10, Ifrd1*, and *Ceacam1* mRNA levels in scramble and *Mettl3*-knockdown MC38 cell lines. (**G**) Western blot analysis of Grb10, Ifrd1, and Mettl3 protein levels in IEC6 and MC38 cell lines with or without *Mettl3* knockdown. Data are presented as mean ± SD. Two-tailed unpaired Student's *t*-test; **P < 0.01; ns, no significance.

To explore the mechanism underlying how *Mettl3* regulates the self-renewal and differentiation of ISCs, RNA-seq was performed to detect gene expression changes in colonic organoids derived from *Mettl3^f/f^ rtTA^+^*and *Mettl3^f/f^* mice with or without Dox treatment. There were 26 differentially expressed genes (DEGs) in Dox-treated *Mettl3^f/f^*group and 1496 DEGs in Dox-treated *Mettl3^f/f^ rtTA^+^* group compared with corresponding DMSO-treated groups, with 7 genes overlapped in both groups that might be induced by Dox treatment (Figure 5A). Therefore, 1489 genes (*P* < 0.05) were differentially expressed in colonic organoids derived from Dox-treated *Mettl3^f/f^ rtTA^+^* group, including 683 downregulated genes and 806 upregulated genes (Supplementary Figure S6A). Interestingly, stem cell marker genes such as *Lgr5* and *Ascl2* (Parikh et al., 2019), TA cell marker genes *Stmn1* and *Tubb5* (Parikh et al., 2019), intestinal development-requiring genes *Cdx2, Notch1*, and *Tm4sf4* (Silberg et al., 2000; Uesaka et al., 2004; Crosnier et al., 2006), the enterocyte marker gene *Alpi*, and the cell proliferation marker gene *Mki67* (encoding Ki67) were all significantly downregulated (Figure 5B and C). Gene Ontology (GO) enrichment analysis revealed that these downregulated genes were enriched in signaling pathways related to cell cycle (Figure 5D). The upregulated genes were mainly enriched in proteoglycans in cancer, cytokine–cytokine receptor interaction, and cytomegalovirus infection pathways based on GO enrichment analysis (Supplementary Figure S6B), which were less related with our phenotypes. To investigate the potential m^6^A target genes, we analyzed m^6^A-RNA immunoprecipitation followed by sequencing (m^6^A-RIP–seq) datasets obtained from human gastrointestinal tract and cell lines (Gao et al., 2020). Three genes, *Grb10, Ifrd1*, and *Ceacam1*, were found among the top 100 upregulated genes in colonic organoids derived from Dox-treated *Mettl3^f/f^ rtTA^+^* group (Figure 5B and C). Further analysis showed that Grb10, Ifrd1, and Ceacam1 were mainly involved in cell growth and differentiation (Supplementary Figure S6C), suggesting that they might be m^6^A targets. Thus, we detected the expression levels of Grb10, Ifrd1, and Ceacam1 in IECs of *Mettl3-*WT and *Mettl3*-KO mice, and qPCR results showed obviously upregulated *Grb10* and *Ifrd1* levels in the *Mettl3*-KO group (Figure 5E). In addition, *Mettl3* knockdown in IEC6 and MC38 cell lines significantly upregulated expression levels of Grb10 and Ifrd1 (Figure 5F and G; Supplementary Figure S7A and B) and inhibited cell growth (Supplementary Figure S7C and D). To verify whether Grb10 and Ifrd1 are specifically expressed in Lgr5^+^ cells, we performed immunofluorescence staining of Grb10 and Ifrd1 in colon sections from *Lgr5^CreERT^* mice. Although at very low levels in the IECs during intestinal homeostasis, Grb10 and Ifrd1 proteins were mainly expressed at the crypt bottom, especially in GFP^+^ Lgr5^+^ stem cells (Supplementary Figure S7E).

Next, we sorted purified m^6^A-deficient Lgr5^+^ stem cells from *Lgr5^CreERT^* mice after treatment with tamoxifen to perform RNA-seq analysis. We found that m^6^A deficiency significantly downregulated the expression of stem cell signature genes and proliferation genes but upregulated the levels of Grb10 and Ifrd1 (Supplementary Figure S8A). GO enrichment analysis identified that loss of m^6^A in Lgr5^+^ stem cell mainly induced dephosphorylation of several signal transduction and inhibited epithelial cell development (Supplementary Figure S8B). Together, these results suggest that m^6^A deficiency suppresses the expression of stem cell signature genes but induces that of Grb10 and Ifrd1 in IECs and Lgr5^+^ stem cells.

### Grb10 and Ifrd1 are the direct targets for m^6^A modification

**Figure 6:**
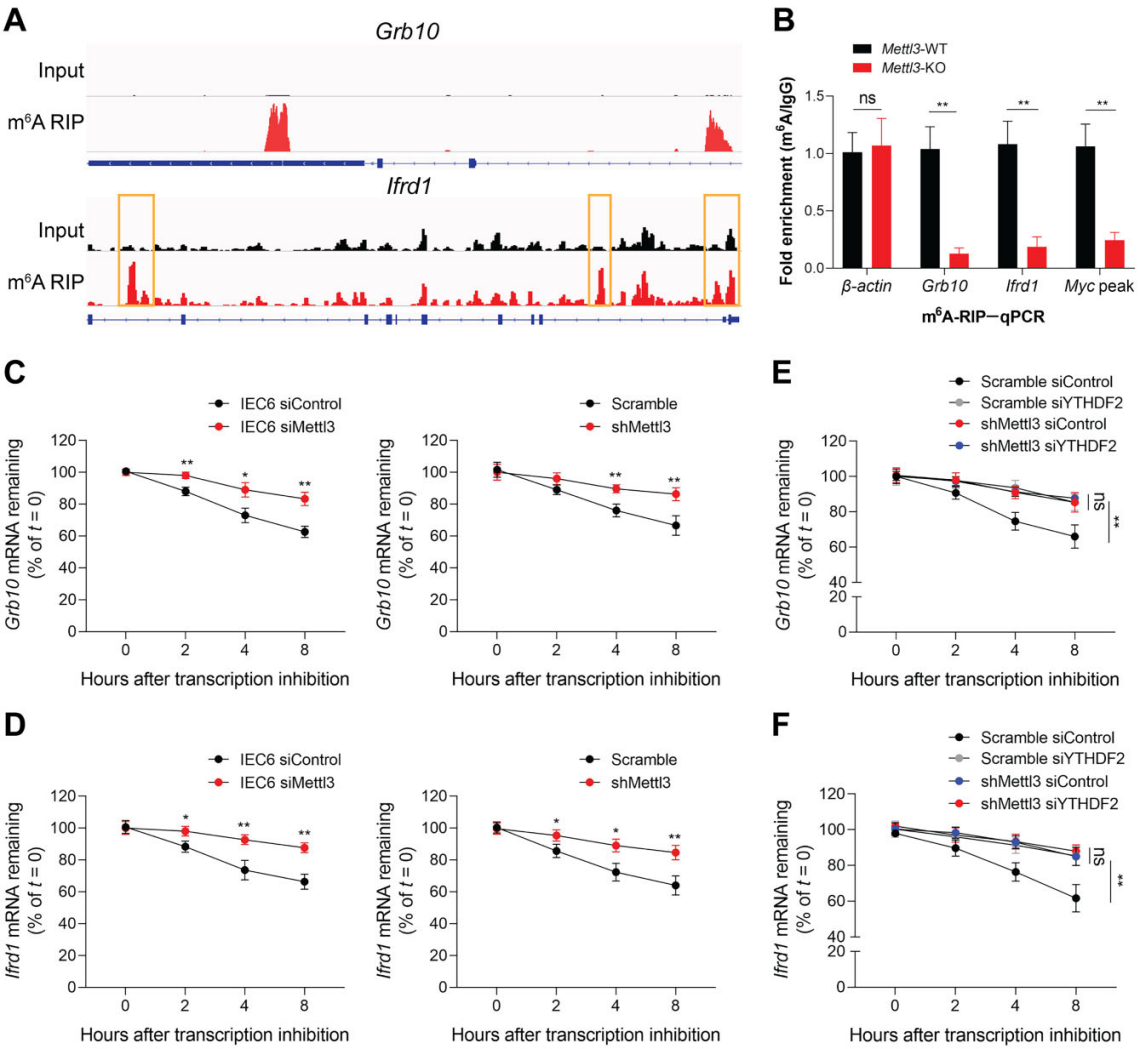
Grb10 and Ifrd1 are the direct targets for m^6^A modification. (**A**) m^6^A-RIP–seq analysis of *Grb10* and *Ifrd1* mRNAs in IECs. (**B**) m^6^A-RIP–qPCR analysis showing m^6^A enrichment on *Grb10* and *Ifrd1* mRNAs in IECs from 2-week-old *Mettl3*-WT but not *Mettl3*-KO mice. Results are presented relative to that obtained with IgG. β*-actin*, m^6^A negative control; *Myc* peak, m^6^A positive control. (**C** and **D**) RNA decay analysis showing *Grb10* (**C**) and *Ifrd1* (**D**) mRNA degradation in IEC6 (left) and MC38 (right) cell lines with or without *Mettl3* knockdown treated with actinomycin D for 2, 4, and 8 h. The residual RNAs were normalized to 0 h. (**E** and **F**) *Grb10* (**E**) and *Ifrd1* (**F**) mRNA degradation in the indicated YTHDF2-silenced *Mettl3*-knockdown MC38 cells treated with actinomycin D for the indicated times. The residual RNAs were normalized to 0 h. Data are presented as mean ± SD. Two-tailed unpaired Student's *t*-test; *P < 0.05; **P < 0.01; ns, no significance.

Analysis of the previous m^6^A-RIP–seq data from human IECs (Gao et al., 2020) indicated a high enrichment and specific m^6^A peaks on *Grb10* and *Ifrd1* mRNAs (Figure 6A). m^6^A-RIP combined with qPCR assays revealed that *Grb10* and *Ifrd1* m^6^A enrichment was markedly reduced in IECs after *Mettl3* knockout (Figure 6B). To further confirm that the deficiency of m^6^A RNA modification may modulate the stability of *Grb10* and *Ifrd1* mRNAs, we performed RNA decay assays. Indeed, *Grb10* and *Ifrd1* transcriptions were more slowly degraded in IEC cell lines after *Mettl3* knockdown (Figure 6C and D). m^6^A mRNA methylation can promote mRNA degradation and is specifically recognized by a family of reader proteins, among which YTHDF2 is responsible for the decay of m^6^A-modified mRNA (Wang et al., 2014). Therefore, we further examined whether *Grb10* and *Ifrd1* mRNA degradation is dependent on m^6^A RNA modification through silencing YTHDF2 expression in stable *Mettl3*-knockdown cell line. As results, Mettl3-mediated m^6^A mRNA modification reduced the stabilization of *Grb10* and *Ifrd1* mRNAs in IECs, which was mainly dependent on YTHDF2 (Figure 6E and F). Taken together, these data suggest that *Mettl3* deletion-mediated decrease in m^6^A modification enhances *Grb10* and *Ifrd1* mRNA stability.

### GRB10 and IFRD1 are upregulated in inflamed colon tissues in mice and humans

To investigate the expression of GRB10 and IFRD1 in human and mouse IBD, we first analyzed the levels in IBD samples based on online databases (GSE31106, GSE179128, and SCP259). As results, GRB10 and IFRD1 levels were significantly upregulated in inflamed tissues compared with healthy controls in both humans and mice (Supplementary Figure S9A–C). Next, we evaluated GRB10 and IFRD1 expression levels in LGR5^+^ stem cells during inflammation based on scRNA-seq database (SCP259). Notably, the expression of GRB10 and IFRD1 was obviously increased in stem cells from IBD patients compared with healthy individuals (Supplementary Figure S9D). Accordantly, METTL3 expression was significantly decreased in stem cells from IBD patients compared with healthy controls (Figure 1E).

**Figure 7:**
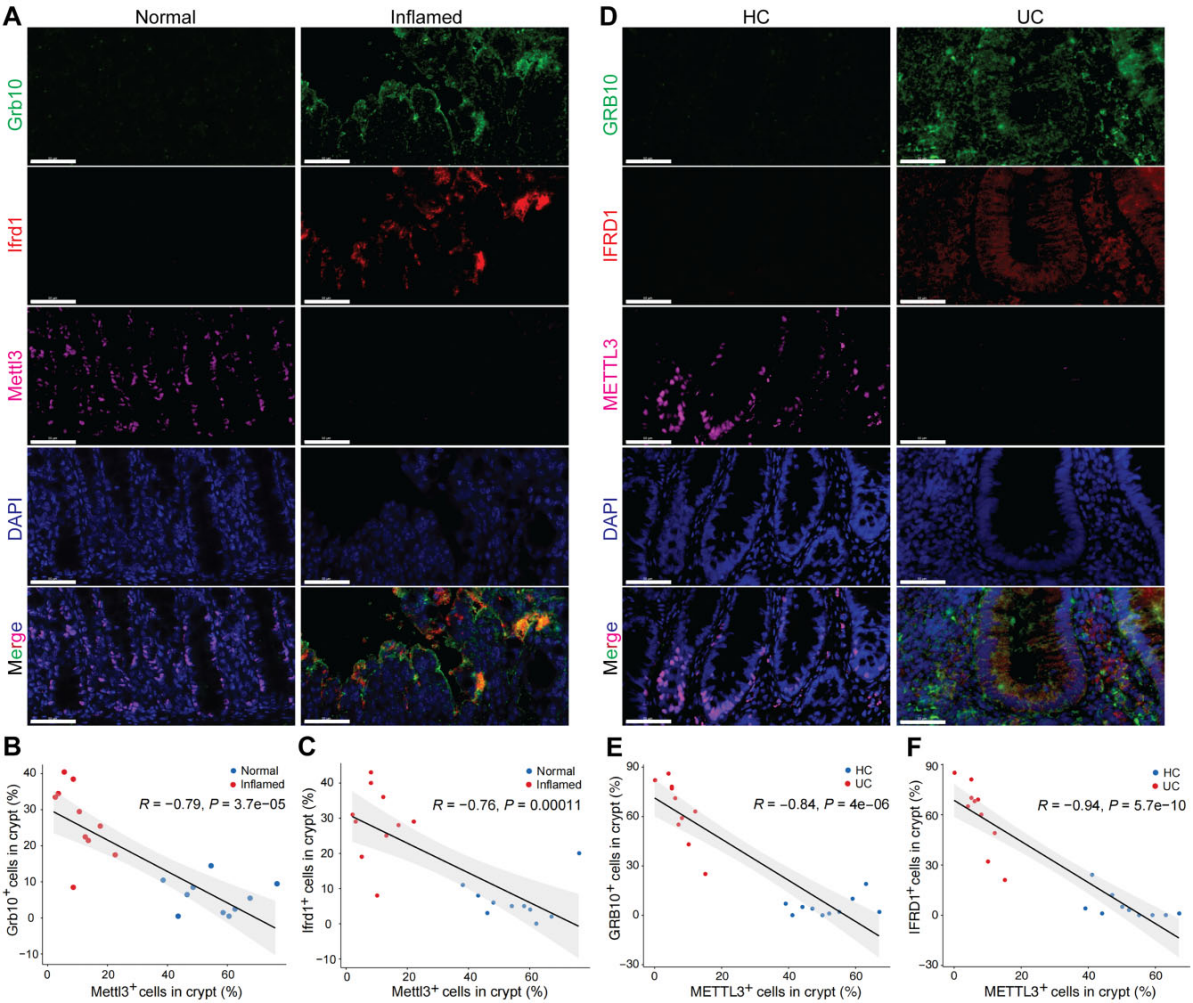
The expression levels of GRB10 and IFRD1 are negatively related to the METTL3 level in either mouse or human inflamed colon tissues. (**A**) Representative images of staining for Grb10, Ifrd1, and Mettl3 in inflamed and normal colons from mice. Scale bar, 50 μm. (**B** and **C**) Spearman's correlation analysis of Grb10 (**B**) or Ifrd1 (**C**) and Mettl3 expression levels in inflamed and normal colons from mice. (**D**) Representative images of staining for GRB10, IFRD1 and METTL3 in colon tissues from UC patients and HC. Scale bar, 50 μm. (**E** and **F**) Spearman's correlation analysis of GRB10 (**E**) or IFRD1 (**F**) and METTL3 expression levels in colon mucosa of HC and UC patients.

Furthermore, we performed immunostaining to detect the levels of GRB10 and IFRD1 in IBD samples and analyzed the correlationship between GRB10 or IFRD1 and METTL3. We found that GRB10 and IFRD1 levels markedly increased in inflamed tissues and were negatively related to METTL3 level in both humans and mice (Figure 7). Taken together, these data demonstrate that Mettl3 and m^6^A mRNA modification modulate the self-renewal and differentiation of ISCs via regulating the stability of *Grb10* and *Ifrd1* mRNAs.

## Discussion

It has been well known that Wnt signaling evokes Notch signaling to modulate the self-renewal and differentiation of stem cell cooperatively, i.e. Wnt signaling in the crypt base maintains the stem-cell proliferative state, and Notch signaling in the TA compartment controls the choice of cell fate (Crosnier et al., 2006; Noah et al., 2011). Gut microbiota plays dual roles during intestinal health and injury, significantly contributing to host intestinal development, functions, and homeostasis (Xing et al., 2020) but exerting pro-inflammatory actions in chronic inflammation and induces dysbiosis (Ni et al., 2017). Mucus constituents, including Muc2, which form a continuous barrier throughout the intestinal tract (Nyström et al., 2021), are secreted by goblet cells that are derived from crypt-residing intestinal Lgr5^+^ stem cells. The functional barrier of mucus layer covering the crypt openings provides an extra barrier against microbial invasion, protecting the stem cell niche at the bottom of the crypt. In the small intestine, epithelial-derived microbial defense mechanisms are largely attributed to the paneth cells; however, in the colon, goblet cells occupying ∼50% in colonic epithelium are high-mucus turnover cells (Nyström et al., 2021). Several observations have correlated a functional mucus barrier defects with the pathogenesis of colitis (Van der Sluis et al., 2006; Carvalho et al., 2012; Johansson et al., 2014). Although previous reports characterized the vital roles of epigenetics in IBD, the role and molecular mechanism of m^6^A methylation, as the most prevalent internal modification in eukaryotic mRNAs (Wei et al., 1975), in intestinal development and inflammation are largely unknown.

Previous studies demonstrated a critical role of m^6^A in maintaining the stemness of ISCs during intestinal regeneration and tumorigenesis (Han et al., 2020; Chen et al., 2021). Our recent work showed that *Mettl14* is essential for the survival of colonic stem cells through regulating NF-κB-mediated anti-apoptotic pathway (Zhang et al., 2022). Here, we showed that METTL3 was decreased in the colon mucosa and stem cells in inflamed colons from mice and humans. Loss of *Mettl3* in IECs or Lgr5^+^ stem cells suppressed the differentiation and self-renewal of ISCs, especially restricted the early differentiation and maturation of goblet cells in colon, and then induced colitis. Our results combined with previous studies have confirmed that m^6^A RNA modification plays a vital role in the survival, self-renewal, and differentiation of ISCs during normal development, inflammation, and tumorigenesis. Notably, a recent study has demonstrated that METTL3 is significantly upregulated in IBD samples, and Mettl3 downregulation attenuates LPS-induced cellular inflammation in mouse IECs *in vitro* and DSS-induced IBD *in vivo* through transfecting lentivirus containing specific shRNA for *Mettl3* (Yang et al., 2022). Additionally, Liu et al. (2023) reported that METTL3 deletion did not affect ISC proliferation, which is inconsistent with our phenotypes. The probable reasons may be the status of UC samples and different experimental tools, e.g. active UC samples without any treatment, specific Cre mice for IECs and intestinal Lgr5^+^ stem cells, and the differences in gut microbiota. Besides, our previously work showed that *Mettl3* deficiency impaired the differentiation of naive T cells, thereby preventing colitis in a lymphopaenic mouse adoptive transfer model (Li et al., 2017). These results strongly indicate that the role of METTL3 in intestinal inflammation is dependent on the cell type involved.

The adaptor protein Grb10 as a cellular partner of tyrosine kinases and signaling substrates mediates several downstream signals (Riedel, 2004), such as insulin, EGF, and Wnt pathways (Tezuka et al., 2007). Grb10 is expected to limit growth, self-renewal, and regeneration of hematopoietic stem cells (Charalambous et al., 2003; Yan et al., 2016). The transcriptional co-repressor Ifrd1 is characterized as an important regulator for Wnt/β-catenin and NF-κB signals (Vietor et al., 2005; Iezaki et al., 2016). It has been known that Wnt, Notch, hedgehog, Eph/ephrin, and tyrosine kinases-related pathways control intestinal stem-cell function and normal intestine development (Sancho et al., 2004; Crosnier et al., 2006; Noah et al., 2011; Hong et al., 2016). Wnt signaling is the first signaling pathway identified to control the gut stem-cell system, and much evidence shows that Wnt pathway is the key factor that maintains stem cells in a proliferative state (Logan and Nusse, 2004; Crosnier et al., 2006). Wnt signaling evokes Notch signaling, which governs the differentiation and cell choice fate in the TA compartment (Crosnier et al., 2006). Therefore, Notch and Wnt signals jointly keep ISC in a proliferating and differentiating state, i.e. neither Wnt pathway activation nor Notch pathway activation is sufficient by itself (Crosnier et al., 2006). Here, we provide new evidence that m^6^A modification directly targets Grb10 and Ifrd1 and maintains the activation of Wnt, Notch, and tyrosine kinases-mediated signals in ISCs. However, how Grb10 and Ifrd1 control these key pathways in the self-renewal and differentiation of ISCs during normal development and inflammation is still unclear. To investigate the proliferating and differentiating transcript and protein profiles of ISCs regulated by Grb10 and Ifrd1 should be a challenging and interesting research direction in the future. In addition, we could not rule out the potential contributions of downregulated targets in regulating self-renewal and differentiation of Lgr5^+^ stem cells due to the complicated role of m^6^A mRNA modification.

In conclusion, this study identifies a key role of *Mettl3* in the self-renewal and differentiation of ISCs during intestinal development and inflammation. Loss of *Mettl3* decreases m^6^A modification on *Grb10* and *Ifrd1* mRNAs to enhance their stability and expression, which then may inhibit Wnt, Notch, and tyrosine kinases-mediated signals in ISCs. Therefore, *Mettl3* and m^6^A RNA modification could serve as potential molecular targets to treat intestinal dysplasia and chronic inflammation.

## Materials and methods

### Participants

All patients with UC were recruited from the Department of Gastroenterology, Ruijin Hospital, Shanghai Jiao Tong University School of Medicine (Shanghai, China) from October 2018 to May 2020. The diagnosis of UC was based on clinical, radiologic, and endoscopic examinations and histologic findings, as described previously (Mosli et al., 2017). Biopsy samples were taken from inflamed or uninflamed regions of the colon from patients with active UC (*n* = 15) and health control individuals without IBD (*n* = 10). The baseline characteristics for UC patients are described in Supplementary Table S1. Healthy volunteers with age and sex matched with UC patients, who signed informed consent, received enteroscopy to confirm that there was no intestinal disease, and normal colons were taken as controls. The disease severity was assessed according to international standard criteria: the Mayo scores for UC patients. The study was approved by the Ethic Committee and Institutional Review Board for Clinical Research of Ruijin Hospital, Shanghai Jiao Tong University School of Medicine (ID: 2019-186). Written informed consent was also obtained from all participants before the study protocol began.

### Mice

*Mettl13^f/f^* mice were generated as previously described with CRISPR–Cas9 technology by insertion of two loxp sites into *Mettl3* genome loci (Henao-Mejia et al., 2016). The gRNAs and donor oligonucleotides for *Mettl3* gene were shown in previous study (Li et al., 2017). *Mettl13^f/f^* mouse was crossed with *Villin-Cre* strain to generate *Villin-Cre^+^; Mettl13^f/f^* conditional knockout mouse. We crossed the Dox-inducible *rtTA-Cre* strain (B6.Cg-Tg(tetO-cre)1Jaw/J × B6.Cg-Gt(ROSA)26Sor^tm1(rtTA*M2)Jae^/J) with *Mettl3^f/f^* mouse to generate *Mettl3^f/f^ rtTA^+^* mouse strain that *Mettl3* can be deleted under the treatment of Dox. *Mettl3^f/f^* mouse was crossed with *Lgr5-eGFP-IRES-Cre^ERT2^* (*Lgr5^CreERT^*) strain to generate *Mettl3^f/f^ Lgr5^C^^reERT^* mouse that *Mettl3* can be deleted under tamoxifen treatment. *Villin-Cre* (B6.Cg strain; Stock No: 003802), B6.Cg-Tg(tetO-cre)1Jaw/J (Stock No: 006234), B6.Cg-Gt(ROSA)26Sor^tm1(rtTA*M2)Jae^/J (Stock No: 006965), and *Lgr5-eGFP-IRES-Cre^ERT2^* (B6.129P2 strain; Stock No: 002120) mice were obtained from Jackson Laboratory and had been backcrossed to C57BL/6N mice for >10 generations. All of the knockout and wild-type mice were sex- and age-matched littermates and co-housed for all experiments described. All the mice were maintained under specific pathogen-free conditions and used in accordance to the animal experimental guidelines set by the Institutional Animal Care and Use Committee (IACUC) of Yale University.

### Tamoxifen treatment

For *Mettl3^f/f^ Lgr5^CreERT^* mice, the tamoxifen-inducible, Cre-mediated recombination results in deletion of the floxed sequences in the Lgr5-expressing cells of the offspring. Tamoxifen was added to corn oil (20 mg/ml) and shaken overnight at 37°C protected from light. Mice were administered 75 mg tamoxifen/kg body weight via intraperitoneal injection daily for 7 days and then rested for 7 days before experimental use.

### DSS-induced colitis analysis

*Mettl3^f/f^ Lgr5^CreERT^*and *Lgr5^CreERT^* littermates were treated with tamoxifen for 7 days and rested for 7 days, and then 2% DSS was added in their drinking water. The weight of mice was measured at the same time point every day. On Day 7, the severity of DSS-induced colitis was detected by endoscopy analysis. Stool, blood vessels, particle size, and translucency were used to evaluate the colitis score, with detailed scoring criteria as described previously (Kodani et al., 2013). Mice were euthanized on Day 9, and then the colon length was detected and colon tissues were collected.

### Lgr5^+^ stem cell organoid culture

The colonic crypts were isolated from *Mettl3^f/f^ Lgr5^CreERT^* and *Lgr5^CreERT^* control mice. Epithelial cells were dissociated with TrypLE express (Invitrogen) and purified Lgr5-GFP^high^ cells were sorted by flow cytometry. To activate Cre^ERT2^, Lgr5-GFP^high^ cells were incubated with a low dose of 4-hydroxytamoxifen (100 nM) for 12 h and cultured in growth medium (Sato et al., 2009). IntestiCult Organoid Growth Medium (Mouse) (STEMCELL, cat. 06005) was use for the establishment and maintenance of Lgr5^+^ single cell organoids, by following the manufacturer's instructions. The entire medium was changed every four days.

### IHC and histological analysis

Mouse intestinal tissue segments were harvested and fixed in 10% formalin solution overnight at room temperature. Post-fixation samples were moved to 70% ethanol and then sent to the Department of Pathology, Yale University School of Medicine, for processing. Samples were embedded in paraffin and sectioned at 5 mm prior to tissue histology. H&E and AB/PAS staining were processed following the manufacturer's instructions. METTL3 staining in colon tissues from UC patients and health controls was scored as follows: 0 (<5% positive cells in IECs), 1 (5%–25% positive cells in IECs), 2 (26%–50% positive cells in IECs), and 3 (>51% positive cells in IECs).

### Permeability of FITC-dextran

Mice were fasted for 12 h and administered FITC-dextran (50 mg/kg). After 4 h, the peripheral blood of mice was collected and centrifuged (3000 r/min, 4°C, 10 min). Then, 100 μl serum was obtained from the supernatant and added to a 96-well plate. Finally, the fluorescence intensity of serum was measured using a fluorescence microplate reader (excitation wavelength: 480 nm; emission wavelength: 520 nm).

### Cell growth assay

MC38 cells were infected with mouse *Mettl3*-specific shRNA lentivirus and negative control shRNA lentivirus. Rat *Mettl3*-specific siRNA oligonucleotides and negative control siRNA were transfected into IEC6 cell line. The transfected and transduced cell lines (*Mettl3*-knockdown and negative control MC38 and IEC6 cell lines) were seeded in 24-well plates at 1 × 10^5^ cells/well and cultured for 5 days. Cell number was counted using a hemocytometer.

### RNA-seq and data processing

Purified m^6^A-deficient and negative control Lgr5-GFP^high^ cells were sorted by flow cytometry from *Lgr5^CreERT^* mice after treatment with tamoxifen. Colonic organoids from *Mettl3^f/f^ rtTA^+^* and *Mettl3^f/f^* mice were treated with Dox or DMSO. Total RNAs were isolated using the RNeasy Micro Kit (QIAGEN, cat. 74034) following the manufacturer's protocol. mRNA for RNA-seq analysis was purified using polyA^+^ selection and RNA-seq libraries were constructed by Yale Center for Genome Analysis (YCGA) using SMARTer® Stranded Total RNA-Seq Kit-Pico Input Mammalian (Clontech). Visualizations using volcano plot and heatmap were produced with R. GO enrichment analysis of biological process was performed by Reactome signaling pathway analysis based on DEGs.

### Bulk RNA-seq data extraction and processing

Two transcription datasets under accession GSE179128 and GSE23597 were downloaded from the Gene Expression Omnibus (GEO) database. The statistical analysis and data visualization were achieved by R (3.6.0).

### scRNA-seq data extraction and processing

The scRNA-seq data under accession SCP259 and GSE186913 were downloaded from the Single Cell Portal database to perform the IEC analyses. Seurat's standard process was conducted. Gene expression was normalized using the default parameter of the Seurat package. The cell clustering and t-SNE visualization were according to the original paper (Smillie et al., 2019). To optimize the gene expression matrix, Markov affinity-based graph imputation of cells (MAGIC) (van Dijk et al., 2018) was used to impute gene expression data.

### CIBERSORTx deconvolution analysis

CIBERSORTx is a computational framework to infer cell-type abundance and cell-type-specific gene expression from RNA profiles of intact tissues (Newman et al., 2019). To utilize this algorithm to deconvolute bulk RNA-seq data, we first derived a cell-type expression matrix from scRNA-seq database (SCP259). This matrix established a benchmark for cell-type proportions in intestinal epithelial tissue. Second, we obtained the bulk RNA-seq data (GSE179128 and GSE23597) and then estimated the proportion of IEC types using CIBERSORTx based on the derived cell-type expression matrix.

### Statistical analysis

Statistical analyses were performed with GraphPad Prism 8.01. Paired or unpaired Student's *t*-test and two-tailed Mann–Whitney *U* test were used for measurement data of two groups. One-way analysis of variance (ANOVA) was used for measurement data of more than two groups. General statistical analysis was calculated with a confidence interval of 95%. *P* ≤ 0.05 was considered statistically significant (*P < 0.05; **P < 0.01; ***P < 0.001; ****P < 0.0001). Data are presented as mean ± SD or SEM as indicated in the figures.

## Supplementary Material

mjae060_Supplemental_File

## Data Availability

The floxed *Mettl3* mouse strain can be provided by Zhengting Wang pending on scientific review and a completed materials transfer agreement. Request for mouse lines should be submitted to Zhengting Wang (zhengtingwang@shsmu.edu.cn). The RNA-seq data used in the study are available in a public repository from NCBI (GSE243214).

## Notes

**Author contributions:** C.D. conceived and designed the experiments. C.D. performed the experiments and wrote the manuscript. X.Y., H.C., and Y.Y. analyzed the RNA-seq data. C.D., X.Y., H.L., H.-B.L., Y.Y., and Z.W. contributed to the manuscript revision and discussion. Y.C. and H.X. performed some experiments. M.R., H.X., H.L., Y.G., and J.Z. contributed to the key discussions and technical support. C.D., W.C., W.H., and Z.W. supervised the entire project.
