# Supplementary material for: METTL3 modulates colonic epithelium integrity via maintaining the self-renewal and differentiation of Lgr5+ stem cell
Source: J Mol Cell Biol. 2025 Jan 6;17(2):mjae060. doi: 10.1093/jmcb/mjae060 (PMC12309382; doi:10.1093/jmcb/mjae060)
Supplement: mjae060_Supplemental_File [file mjae060_supplemental_file.pdf]

Supplementary material

METTL3 modulates colonic epithelium integrity via maintaining the self-renewal and differentiation of $Lgr5^+$ stem cell

Chenbo Ding[1,2,3†,*], Xinhui Yang[4,†], Hua Liu[4,†], Manolis Roulis[3], Huifang Chen[2], Yunzhu Chen[2], Hao Xu[3], Yimeng Gao[5,6], Jie Zhong[4], Hua-Bing Li[1,2,3], Youqiong Ye[2], Wei Cai[7,*], Weiguo Hu[7,*], and Zhengting Wang[4,*]

[1] Institute of Immunological Innovation & Translation, Chongqing Medical University, Chongqing 400016, China

[2] Shanghai Institute of Immunology, State Key Laboratory of Oncogenes and Related Genes, Shanghai Jiao Tong University School of Medicine, Shanghai 200025, China

[3] Department of Immunobiology, Yale University School of Medicine, New Haven, CT 06520-8055, USA

[4] Department of Gastroenterology, Ruijin Hospital, Shanghai Jiao Tong University School of Medicine, Shanghai 200025, China

[5] Section of Hematology, Yale Cancer Center and Department of Internal Medicine, Yale University School of Medicine, New Haven, CT 06520-8055, USA

[6] Institute for Regenerative Medicine, Shanghai East Hospital, School of Life Sciences and Technology, Tongji University, Shanghai 200123, China

[7] Department of General Surgery, Shanghai Minimally Invasive Surgery Center, Shanghai Institute of Immunology, Ruijin Hospital, Shanghai Jiao Tong University School of Medicine, Shanghai 200025, China

[†] These authors contributed equally to this work.

* Correspondence to: Zhengting Wang, Tel.: +86-21-64370045, E-mail: zhengtingwang@shsmu.edu.cn; Chenbo Ding, E-mail: chenbo.ding@cqmu.edu.cn; Weiguo Hu, E-mail: wghu@rjh.com.cn; Wei Cai, E-mail: caiwei@shsmu.edu.cn

Supplementary Materials and methods

Isolation of intestinal epithelial cells

For the isolation of colonic epithelial cells, the colon tissue was cut into 2 mm segments in RPMI-1640 medium containing 2.5 mM EDTA and 2% fetal bovine serum (FBS), and then incubated on a rocker for 30 min at 4°C. Epithelial cells were released by vigorous shaking twice and passed through a 70-μm strainer, and then washed with cold DPBS containing 2% FBS.

BrdU analysis

The BrdU analysis was performed as previously described (Roulis et al., 2020). Briefly, intraperitoneally injected BrdU at a dose of 100 μg per g of body weight 2 hours before mice were euthanized. For the colonic organoid culture, 10 mg/ml BrdU added in growth media at a concentration of 50 umol/L, incubated 2 hours at 37°C and 5% CO_2. BrdU immunofluorescence staining was performed in formalin fixed paraffin-embedded colon tissues or organoids with the BrdU antibody (Biolegend, cat. 364108). E-cadherin staining was performed with FITC mouse anti-E-cadherin (BD Transduction Laboratories, cat. 612130), and was used to better identify the structure of crypt. Confocal imaging was conducted with a Nikon Eclipse Ti microscope equipped with UltraVox spinning disk (PerkinElmer) and data was analyzed using the Volocity Legacy 6.0.1 software (PerkinElmer). The number of $BrdU^+$ cells per crypt was counted in a blinded fashion.

Isolation of colonic crypts and organoid culture

IntestiCult Organoid Growth Medium (Mouse) (STEMCELL, cat. 06005) was use for the establishment and maintenance of mouse colonic organoids, and processed following the manufacturer's instructions. Briefly, Mice were sacrificed and about 6 cm colon was harvested from each mouse. The colon was flushed gently with cold PBS containing 1% FBS and cut into 2 mm pieces. A 10 ml serological pipette was used to wash the colonic pieces by pipetting up and down 3 times. Let colonic pieces settle by gravity and carefully removed supernatant. This wash procedure was repeated 15-20 times or until supernatant was clear. Then, the colonic pieces were suspended in 25ml Gentle Cell Dissociation Reagent (STEMCELL, cat. 07174) in a shaker at 20 rpm for 20 min at room temperature. Let colonic pieces settle by gravity for approximately 30 sec and carefully removed supernatant, and resuspended in 10 ml cold PBS containing 0.1% bovine serum albumin (BSA). Pipetted colonic pieces up and down 3 times, the supernatant was removed and passed through a 70μm strainer into a 50ml conical tube. Repeated this procedure 6 times, and collected the crypts. The quality of the suspensions was assessed by using an inverted microscope, and counted the crypt number. Crypts were washed by centrifugation at 200g and then used for organoid development in domes made by Matrigel (Corning, cat. 356231) and IntestiCult Organoid Growth Medium (1:1). 50 μl suspension for each dome was pipetted into 24-well plate. Next, the plate was incubated at 37°C for 10-15 min until the Matrigel was solidified. Finally, 750 μl complete IntestiCult Organoid Growth Medium was added to each well and incubated at 37°C and 5% CO_2. For colonic

crypts isolated from *Mettl3*$^{f/f}$ *rtTA*$^{+}$ mouse, organoids culture were added Dox at day0, and replaced entire medium without Dox at day5.

RNA isolation and qRT-PCR

TRIzol reagent (Invitrogen) was added to colonic epithelial cells, IEC6 and MC38 cell lines and then processed following the manufacturer's instructions. RNA was further purified using the RNase-Free DNase Set (QIAGEN, cat. 79256). The RNA isolation of organoids was performed with RNeasy Micro Kit (QIAGEN, cat. 74034) following the manufacturer's protocol. Maxima H Minus Reverse Transcriptase Kit (ThermoFisher Scientific, cat. EP0753) was used for cDNA synthesis. Sigma KiCqStart predesigned SYBR green primers and iTaq Universal SYBR Green Supermix were used for real time PCR, and *β-actin* mRNA level was used as internal control to calculated mRNA relative abundance. Primer sequences used for qPCR are listed in Supplementary Table S2.

Colon lymphocytes isolation

Colon tissues was harvested as described and kept in cold RPMI medium until next procedure, when they were turned inside out and incubated in 20 ml digestion medium (RPMI with 0.5mg/ml Dispase, 1mg/ml Type II Collagenase and 2% FBS). For the digestion procedure, tissues were treated for 50 minutes at 37℃, with 600rpm of stirring speed. When digestion was over, the samples were filtered through 70 μm cell strainer, and rinsed with additional 25 ml of FACS buffer (DPBS containing 2% FBS), followed by centrifuging at 500g for 10 minutes. The cell pellet was collected and washed with FACS buffer, and lymphocytes were purified using 40% percoll, and then were strained to remove any undigested material and washed in FACS buffer before processing for flow cytometry analysis.

Flow cytometry

For staining of surface markers and nuclear factors was performed as previously described (Ding et al., 2022). Data were analyzed by using FlowJo software (version 9.0 or higher, BD Bioscience). The list of antibodies and reagents used in flow cytometry is shown in Supplementary Table S3.

Immunofluorescence analysis

Collected colonic tissue samples, and colonic organoids derived from whole colonic crypts in WT and KO groups from 2-week old littermates. Formalin-fixed paraffin-embedded tissue sections were dewaxed 1 hour at 60°C incubator, gradient ethanol hydration, washed and antigen retrieval was performed by microwave heating in citrate buffer. Washed with PBS 3 times, and then rinsed with PBS containing 0.5% Triton X100 and 0.05% Tween-20 10 min. And all samples were added in blocking buffer (PBS+0.025% Triton X100, 0.05% Tween-20, and 5% BSA) 30 min at room temperature. BrdU immunostaining was performed with the BrdU antibody (Biolegend, cat. 364108) overnight at 4°C. Primary antibodies included rabbit anti-MUC2 (Santa Cruz H-300) at 1:200 dilution, overnight at 4°C with an anti-rabbit Alexa Fuor 594 secondary antibody at a 1:1,000 dilution, for 1

hour at room temperature. Immunostaining for UEA1 was performed with FITC mouse anti-UEA1 (Vector Laboratories, cat. L-1060) overnight at 4°C. Immunostaining for E-cadherin was performed with FITC mouse anti-E-cadherin (BD Transduction Laboratories, cat. 612130) at a 1:500 dilution, overnight at 4°C. Confocal imaging was conducted with a Nikon Eclipse Ti microscope equipped with UltraVox spinning disk (PerkinElmer) and data was analyzed using the Volocity Legacy 6.0.1 software (PerkinElmer). The numbers of BrdU or Muc2 and UEA1 immunostaining positive cells for per well-oriented crypt or crypt-villus unit were quantified in a blinded fashion. Each samples, the visual field of different positions was selected, and counted at least ten crypts in each field, then calculated the average percentage of positive cells per crypt.

***siRNA transfectio*n and lentivirus infection**

Rat *Mettl3* specific siRNA oligonucleotides and negative control siRNA were transfected into IEC6 cell line. For lentiviral infection, MC38 cells were infected with mouse *Mettl3* specific shRNA and negative control shRNA lentiviral vectors for 12 hours. Then, the culture medium was removed and fresh medium was added; 2 days post-infection, stable pools of cells were selected with 3 μg/ml puromycin (Invitrogen, USA) for 8 days. Stable knockdown cell lines were generated as previously described (Liu et al., 2012). In addition, mouse YTHDF2 specific siRNA oligonucleotides (Cat. no. AM16708, Assay ID 144389, Invitrogen, USA) and negative control siRNA (Cat. no. AM4611, Invitrogen, USA) were transfected into stable *Mettl3* knockdown MC38 cell line. Lipofectamine RNAiMAX (Invitrogen, USA) in OptiMEM (Invitrogen, USA) was used in siRNA transfection and following the manufacturer's protocol.

Western blot

Total protein of MC38 cells was extracted with RIPA lysis buffer supplemented with protease inhibitors (ThermoFisher Scientific). Antibodies against *Mettl3*, *Grb10*, *Ifrd1* and *β-actin* (Cell Signaling Technology, cat. 4970) were used at a 1:1,000 dilution in 5% no-fat milk buffer at 4°C overnight. 0.1% PBST buffer washed the membranes three times, 10 min each time. After that, an HRP-conjugated secondary antibody (Cell Signaling Technology, cat. 7074) was added to the membranes and incubated at room temperature for 1 hour. After extensive wash, the signal was detected by enhanced chemiluminescence with pico ECL using Chemidoc MP (Biorad).

m^{6}A-RIP–qPCR

m^{6}A-RIP–qPCR analysis was conducted as described previously (Liu et al., 2019). Briefly, Total RNA was isolated from IECs. Polyadenylated RNA was further enriched from total RNA by using Dynabeads® mRNA Purification Kit (Invitrogen) and incubated with anti-m^{6}A antibody (Synaptic System, cat. 202003) or rabbit IgG in RIP buffer (150 mM NaCl, 0.1% NP-40, and 10 mM Tris-HCl) for 2 hours at 4°C. The mixture was then immunoprecipitated by incubation with 50 μl protein-A beads (Sigma, P9424) at 4°C for an additional 2 hours. After extensive wash with RIP buffer, bound

RNA was eluted by m^{6}A nucleotide by using TRIzol reagent (Invitrogen), and then purified by 75 % ethanol precipitation. Maxima H Minus Reverse Transcriptase Kit (ThermoFisher Scientific, cat. EP0753) was used for cDNA synthesis. The qPCR was conducted and the expression of *Grb10* and *Ifrd1* was normalized to the input sample. *β-actin* as m^{6}A negative control, *Myc* peak as m^{6}A positive control (Li et al., 2017).

RNA decay analysis

Stable *Mettl3* knockdown MC38 cells and negative control cells, and stable *Mettl3* knockdown MC38 cells with YTHDF2 specific siRNA transfection were treatment with actinomycin D (5 μg/ml) (Sigma-Aldrich, cat. A1410) for 0, 2, 4 and 8 hours, and then collected and subjected to RNA extraction. Total RNA was isolated and qPCR was conducted for mRNA levels as described above.

Supplementary References

Ding, C., Xu, H., Yu, Z., et al. (2022). RNA m6A demethylase ALKBH5 regulates the development of γδ T cells. Proc Natl Acad Sci U S A *119*, e2203318119.

Li, H.B., Tong, J., Zhu, S., et al. (2017). m^{6}A mRNA methylation controls T cell homeostasis by targeting the IL-7/STAT5/SOCS pathways. Nature *548*, 338–342.

Liu, X., Ma, B., Malik, A.B., et al. (2012). Bidirectional regulation of neutrophil migration by mitogen-activated protein kinases. Nat Immunol *13*, 457–464.

Liu, Y., You, Y., Lu, Z., et al. (2019). N^6-methyladenosine RNA modification-mediated cellular metabolism rewiring inhibits viral replication. Science *365*, 1171–1176.

Roulis, M., Kaklamanos, A., Schernthanner, M., et al. (2020). Paracrine orchestration of intestinal tumorigenesis by a mesenchymal niche. Nature *580*, 524–529.

Supplementary Figures

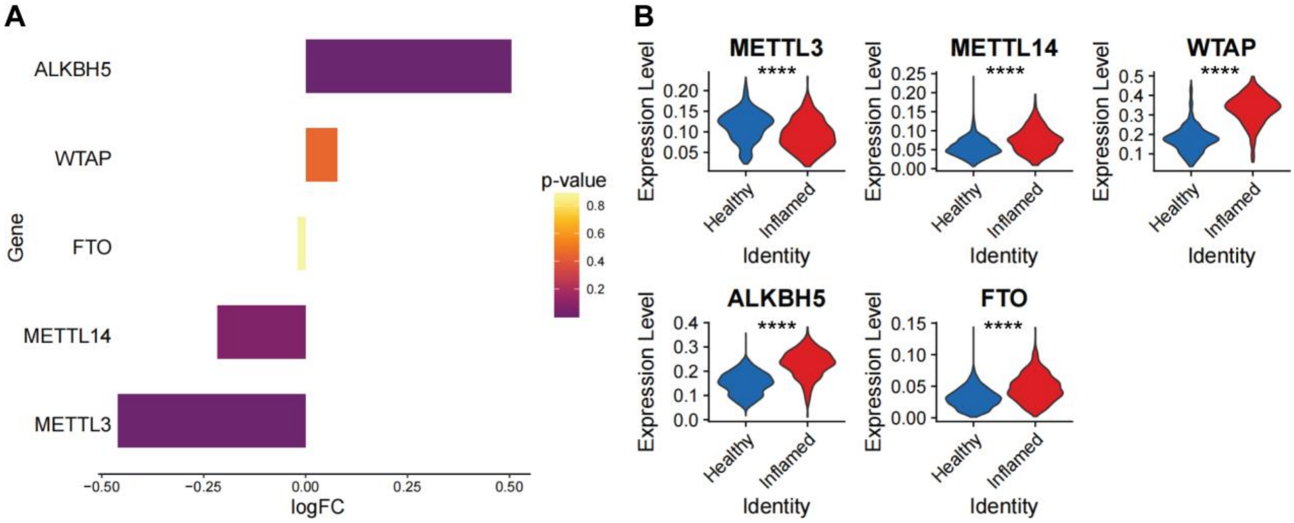

Supplementary Figure S1. The expression m^{6}A binding proteins in inflamed colon tissues and intestinal stem cells. Related to Figure 1.

(**A**) The expression of m^{6}A binding proteins in inflamed patients based on bulk RNA-seq database (GSE179128).

(**B**) The expression of m^{6}A binding proteins in intestinal stem cells from health and inflamed patients based on scRNA-seq database (SCP259).

Data are presented as mean ± SD. Two-tailed unpaired Student's *t*-test; ****$P < 0.0001$.

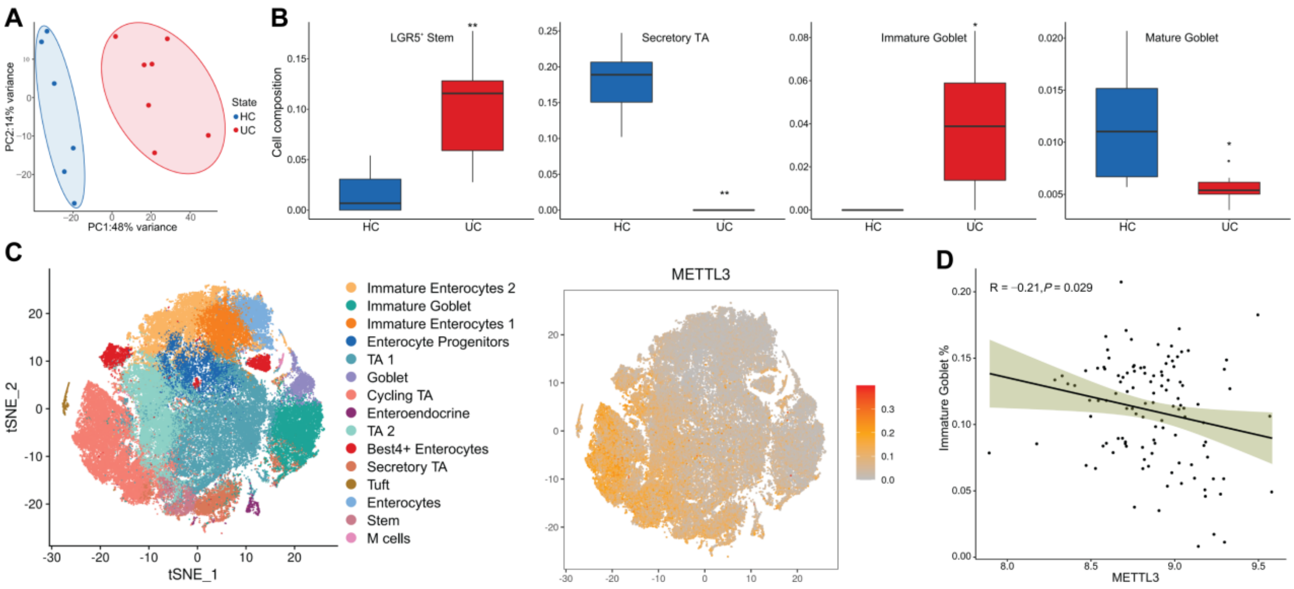

Supplementary Figure S2. METTL3 may be associated with the development of goblet cell. Related to Figure 1.

(**A**) PCA analysis of health controls (HC) and ulcerative colitis (UC) patients based on bulk RNA-seq database (GSE179128).

(**B**) Cell composition of LGR5^{+} stem cell, secretory TA, immature goblet and mature goblet cells estimated by CIBERSORTx from HC and UC patients based on bulk RNA-seq database (GSE179128).

(**C**) *t*-SNE plot with clustering results and METTL3 expression in intestinal epithelial cells based on scRNA-seq database (SCP259).

(**D**) Spearman's correlation analysis of METTL3 expression and the proportion of immature goblet cells estimated by CIBERSORTx in UC patients based on bulk RNA-seq database (GSE23597).

Data are presented as mean ± SD. Wilcoxon test; *$P < 0.05$; **$P < 001$.

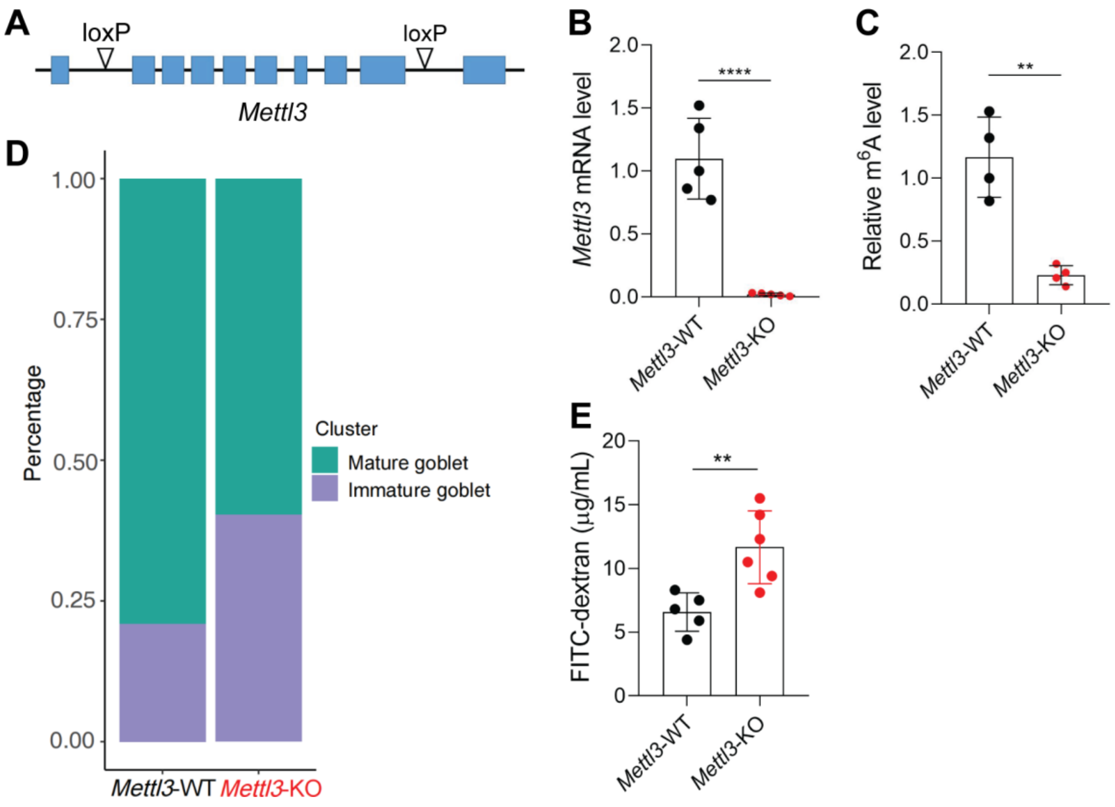

Supplementary Figure S3. Generation and verification of *Mettl3*-KO mice in intestinal epithelial cells. Related to Figure 2.

(**A**) Two loxP sites were inserted into the first and last introns of *Mettl3* by CRISPR technology.

(**B**) Verification of *Mettl3* depletion in IECs isolated from *Mettl3*-WT (n = 5) and *Mettl3*-KO littermates (n = 5) by qRT-PCR analysis.

(**C**) The relative m^6A level in IECs isolated from *Mettl3*-WT (n = 4) and *Mettl3*-KO littermates (n = 4) by m^6A dot blot analysis.

(**D**) The analysis of the percentage of mature and immature goblet cells in *Mettl3*-WT and *Mettl3*-KO mice based on the publicly available scRNA-seq data (GSE186913).

(**E**) Permeability assay of *Mettl3*-WT (n = 5) and *Mettl3*-KO (n = 6) mice via FITC-Dextran.

Data are presented as mean ± SD. Two-tailed unpaired Student's *t*-test; $^{**}P < 0.01$; $^{****}P < 0.0001$.

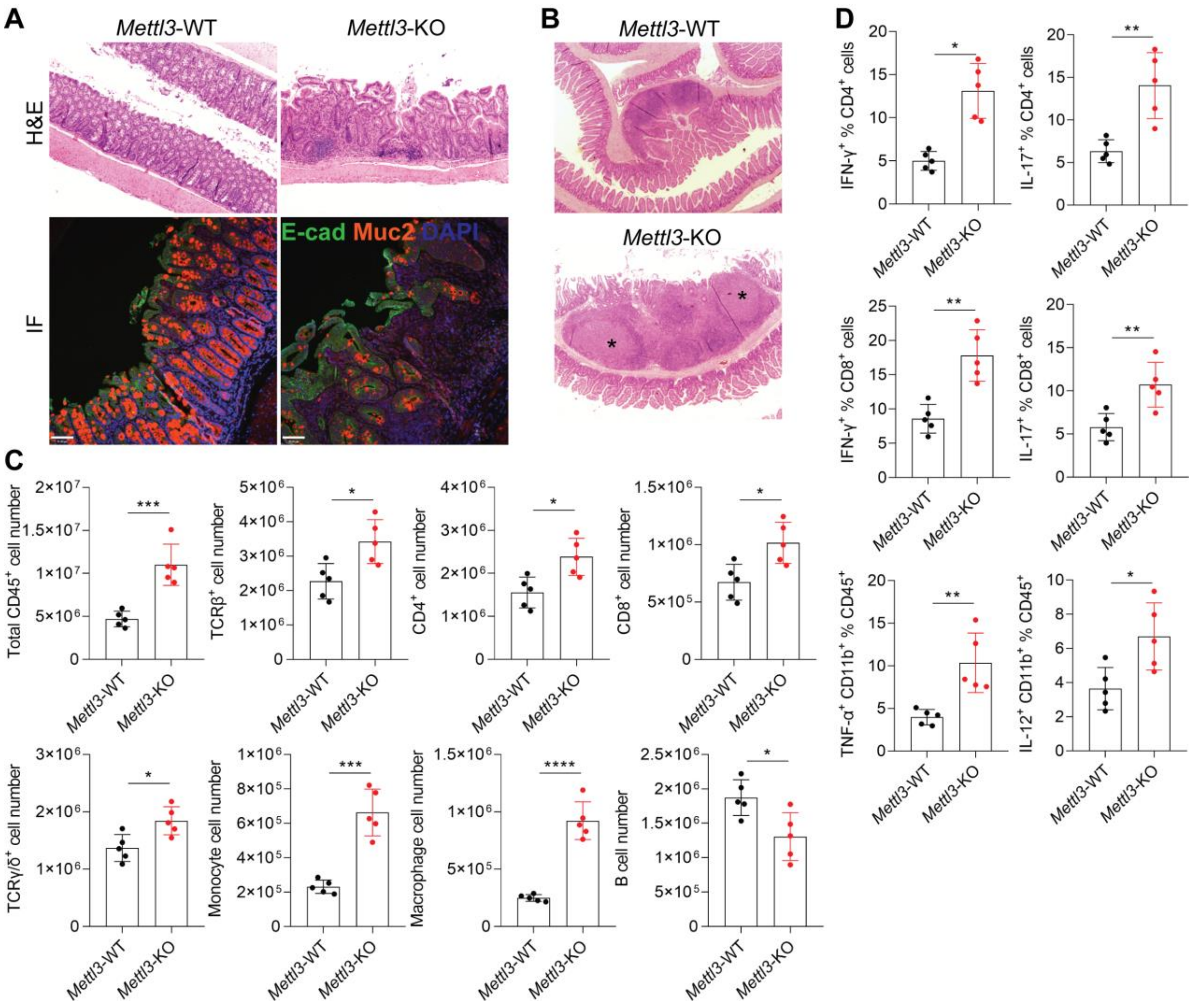

Supplementary Figure S4. *Mettl3* depletion in IECs induces spontaneous inflammation, immune cell infiltration and inflammatory cytokines. Related to Figure 2.

(**A**) Representative H&E (up) and immunofluorescence (IF) (down) staining of distal colon sections obtained from *Mettl3*-WT and *Mettl3*-KO mice at eight-week old.

(**B**) Representative H&E staining of small intestine sections obtained from *Mettl3*-WT and *Mettl3*-KO at eight-week old. The enlargement of Peyer's patches with formation of germinal centers (asterisks).

(**C**) Statistical analysis of total CD45$^+$, TCRβ, TCRγ/δ, CD4$^+$, CD8$^+$, monocyte, macrophage and B cells in colon tissues from *Mettl3*-WT (n = 5) and *Mettl3*-KO (n = 5) mice at eight-week old.

(**D**) Statistical analysis of the percentage of IFN-γ$^+$ or IL-17$^+$ in CD4$^+$/CD8$^+$ lymphocytes, and the percentage of TNF-α$^+$ CD11b$^+$ or IL-12$^+$ CD11b$^+$ in CD45$^+$ cells in colon tissues from *Mettl3*-WT (n = 5) and *Mettl3*-KO (n = 5) mice at eight-week old.

Data are presented as mean ± SD. Two-tailed unpaired Student's *t*-test; **P* < 0.05; ***P* < 0.01; ****P* < 0.001; *****P* < 0.0001.

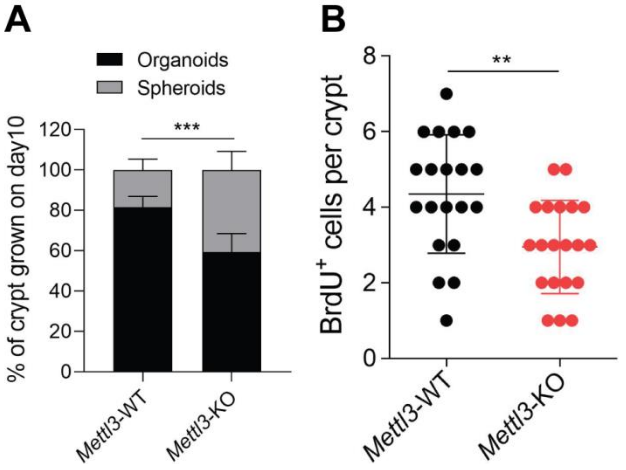

Supplementary Figure S5. Loss of m^{6}A suppresses the growth and self-renewal of intestinal stem cells. Related to Figure 3.

(**A**) Statistical analysis of the balance of organoids and spheroids derived from two-week old *Mettl3*-WT (n = 5) and *Mettl3*-KO (n = 5) mice on cultured day 10.

(**B**) Statistical analysis of crypt proliferation of colonic organoids labelled by BrdU, derived from *Mettl3*-WT (n = 5) and *Mettl3*-KO (n = 5) mice at two-week old.

Data are presented as mean ± SD. Two-tailed unpaired Student's *t*-test; $^{**}P < 0.01$; $^{***}P < 0.001$.

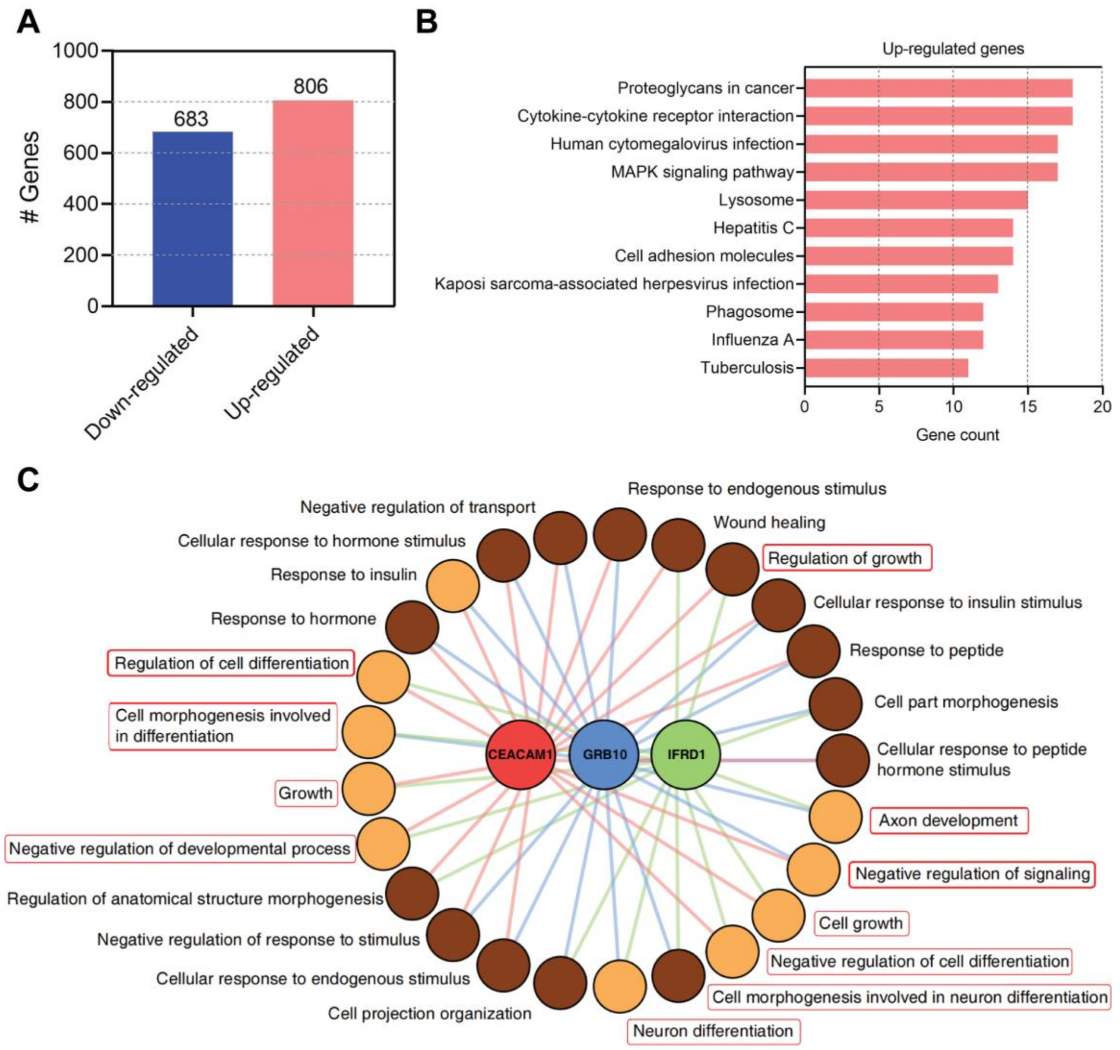

Supplementary Figure S6. Transcriptomic changes in *Mettl3* deficient colonic organoids. Related to Figure 5.

(**A**) Numbers of genes that are up-regulated and down-regulated in Mettl3 deficient colonic organoids. Differently expressed genes based on *P*-value ($P < 0.05$).

(**B**) Gene Ontology (GO) enrichment analysis of biological process for up-regulated genes in Mettl3 deficient colonic organoids. Data represent three independent experiments combined.

(**C**) Network diagram illustrates the GO biological process terms associated with the three genes: Ceacam1 (red), Grb10 (blue), and Ifrd1 (green). The outer circles represent the various biological processes that these genes are involved in, with lines connecting each gene to its associated processes.

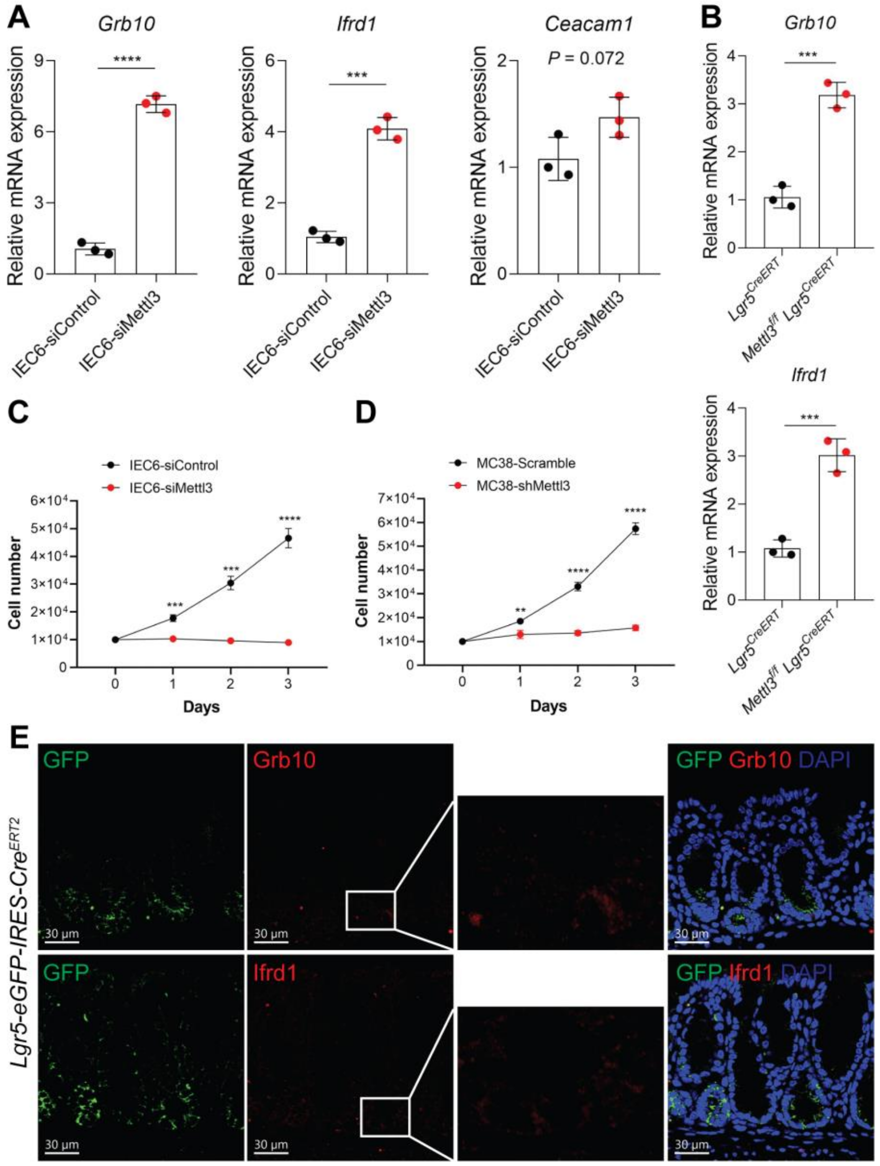

Supplementary Figure S7. *Mettl3* knockdwon induced the level of Grb10 and Ifrd1, while inhibits cell growth in IEC cell lines. Related to Figure 5.

(**A**) qRT-PCR analysis of *Grb10*, *Ifrd1,* and *Ceacam1* mRNAs in negative control and *Mettl3* knockdown IEC6 cell lines.

(**B**) qRT-PCR analysis of *Grb10* and *Ifrd1* mRNAs in $Lgr5^+$ stem cells from $Mettl3^{f/f}$ $Lgr5^{CreERT}$ (n = 3) and $Lgr5^{CreERT}$ (n = 3) mice treated with tamoxifen daily for 7 days and rested for 7 days.

(**C**) Cell growth analysis of IEC6 cell lines transfected with *Mettl3* specific siRNA oligonucleotides or negative control siRNA.

(**D**) Cell growth analysis of MC38 cell lines post infection with *Mettl3* specific shRNA and negative control shRNA lentiviral vectors.

(**E**) Representative IF staining of Grb10, Ifrd1, and GFP in colon sections obtained from $Lgr5^{CreERT}$ mice.

Data are presented as mean ± SD. Two-tailed unpaired Student's *t*-test; $^{**}P < 0.01$; $^{***}P < 0.001$; $^{****}P < 0.0001$.

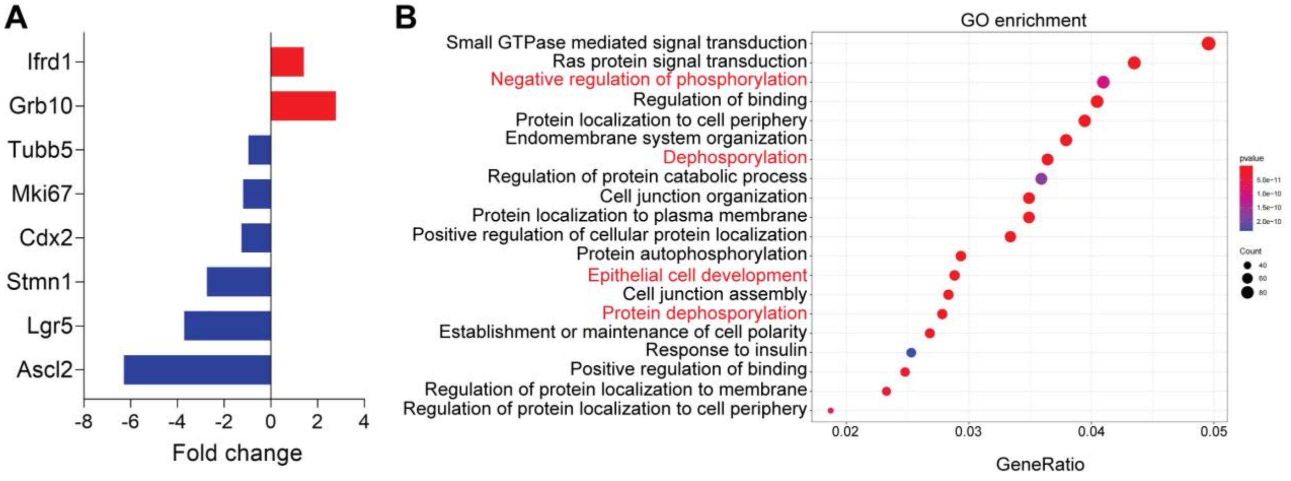

Supplementary Figure S8. m^{6}A deficiency upregulates the expression of Grb10 and Ifrd1, while reduces stem cell signatures in Lgr5$^+$ stem cells. Related to Figure 5.

(**A**) Differentially expressed genes, such as stem cell signatures Ascl2 and Lgr5, TA cell markers Stmn1 and Tubb5, intestinal development required markers Cdx2 and Ki67, Grb10 and Ifrd1 were identified in m^{6}A deficiency Lgr5$^+$ stem cells.

(**B**) Gene Ontology (GO) enrichment analysis of biological process for differentially expressed genes in m^{6}A deficient Lgr5$^+$ stem cells.

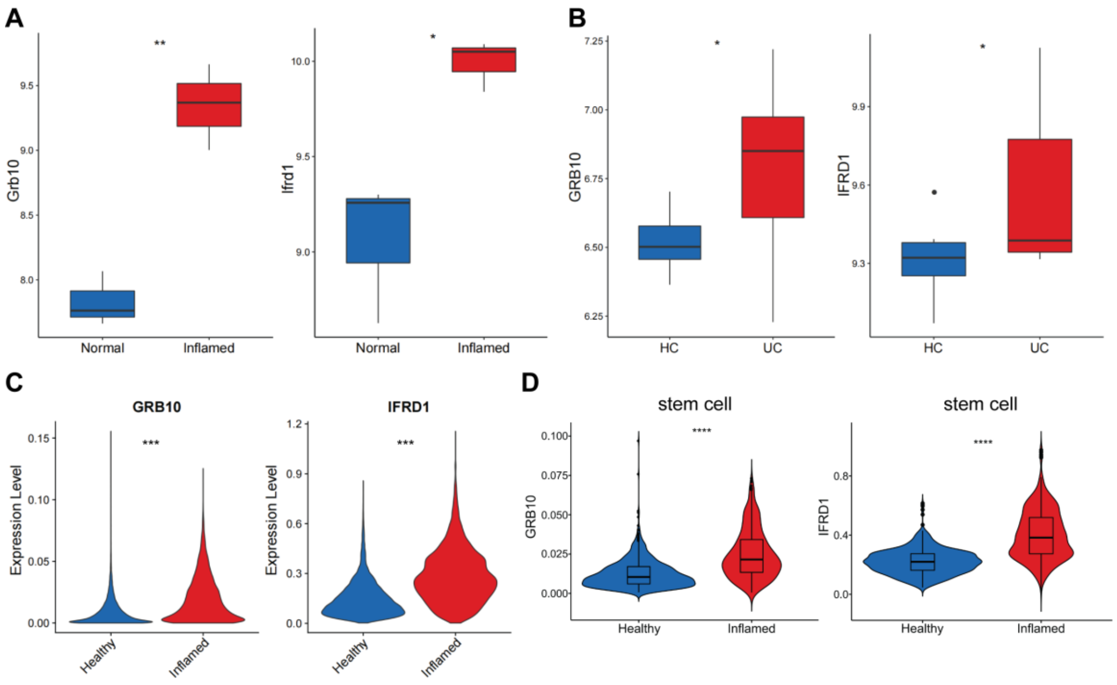

Figure S9. GRB10 and IFRD1 were markedly increased in inflamed colon tissues in mice and humans. Related to Figure 7.

(**A**) Grb10 and Ifrd1 expression in inflamed and normal colons from mice based on bulk RNA-seq database (GSE31106).

(**B**) GRB10 and IFRD1 expression in colon mucosa of ulcerative colitis (UC) and health controls (HC) based on bulk RNA-seq database (GSE179128).

(**C**) GRB10 and IFRD1 expression in intestinal epithelial cells from inflamed and health humans based on scRNA-seq database (SCP259).

(**D**) GRB10 and IFRD1 expression in $LGR5^{+}$ stem cells from IBD patients and health individuals based on scRNA-seq database (SCP259).

Data are presented as mean ± SD. Wald test (**A**, **B**) or two-tailed unpaired Student's *t*-test (**C**, **D**); *$P < 0.05$; **$P < 0.01$; ***$P < 0.001$; ****$P < 0.0001$.

Supplementary Tables

Supplementary Table S1. Clinical characteristics of patients with active UC.

	**UC**
**Samples**	Colon
**Number of patients**	15
**Age (years)**	35.53 ± 12.78
**Gender**	
Male	10
Female	5
**Current therapy**	
5-aminosalicylates	0
Immunosuppressants	0
Biologics	0
Nutritional therapy	0
**Disease extent**	
E1	3
E2	7
E3	5

Supplementary Table S2. Primer sequences used in qRT-PCR analysis.

Gene	Primer sequence (5′ to 3′)
Rat Actin-F	CACTATCGGCAATGAGCGGTTCC
Rat Actin-R	ACTGTGTTGGCATAGAGGTCTTTACG
Rat Grb10-F	TGATTCACAGGACTCAGCATTGGTTC
Rat Grb10-R	TCCACGAGACCTTGTTGCTTGATG
Rat Ifrd1-F	GGCGAATCTTTGGCACTTCTGTTTG
Rat Ifrd1-R	GCACGGTGTTTATTTCCATCTGTAGC
Rat Ceacam1-F	TAGCAGGCAGCAGAGACTATGGAG
Rat Ceacam1-R	TGACTTGGGCAGTGGTGAGAGG
Rat Mettl3-F	CGTAACCTATGCTGACCACTCCAAG
Rat Mettl3-R	TGTGATAGTCCCTGCTGCCTCTC
Mouse Mettl3-F	GAAACAGCTGGACTCGCTTC
Mouse Mettl3-R	GGCACGGGACTATCACTACG
Mouse Grb10-F	GGCATGAGAGTAGCAGACAGGATTG
Mouse Grb10-R	TGGGTGGTATCAAGAAAGGGAAATGTG
Mouse Ifrd1-F	GGCGAATCTTTGGCACTTCTGTTTG
Mouse Ifrd1-R	GCACGGTGTTTATTTCCATCTGTAGC
Mouse Ceacam1-F	ACTTCAATTCCCAGCAACCCAACC
Mouse Ceacam1-R	CAGCAGGACAGACGGACAGATTATG
Mouse Actin-F	AGTGTGACGTTGACATCCGT
Mouse Actin-R	GCAGCTCAGTAACAGTCCGC
Mouse Myc peak-F	GCTTCGAAACTCTGGTGCAT
Mouse Myc peak-R	AATTCCAGCGCATCAGTTCT
Mouse Ythdf2-F	ATAGGAAAAGCCAATGGAGGG
Mouse Ythdf2-R	CCAAAAGGTCAAGGAAACAAAG
Mouse Lgr5-F	CCTACTCGAAGACTTACCCAGT
Mouse Lgr5-R	GCATTGGGGTGAATGATAGCA
Mouse Clca1-F	AGGAAAACCCCAAGCAGTG
Mouse Clca1-R	GCACCGACGAACTTGATTTT
Mouse Creb314-F	GAGCTGGGATTCAACGGTCC
Mouse Creb314-R	CATAGACAACCTCATAGAGGGCA
Mouse Muc2-F	AACTGAATCCTCGACGCCTG
Mouse Muc2-R	TTGGCCCTGTTGTGGTCTTT
Mouse Dclk1-F	CTGGGTTAATGATGATGGTCTCC
Mouse Dclk1-R	TCCTGGTTGTTGGTAGTAGTCC
Mouse Alpi-F	AGGACATCGCCACTCAACTC
Mouse Alpi-R	GGTTCCAGACTGGTTACTGTCA
Mouse ChgA-F	CGATCCAGAAAGATGATGGTC
Mouse ChgA-R	CGGAAGCCTCTGTCTTTCC
Mouse ChgB-F	CCTCTCAAATGCCCTATCCA
Mouse ChgB-R	CACCTTTGACCTCTTTTCCACT
Mouse Tnf-α-F	TCTTCTCATTCCTGCTTGTGG
Mouse Tnf-α-R	GGTCTGGGCCATAGAACTGA
Mouse Il1β-F	TGCTGGTGTGTGACGTTCCC
Mouse Il1β-R	TGAGGCCCAAGGCCACAGGTA
Mouse Il6-F	GTTCTCTGGGAAATCGTGGA
Mouse Il6-R	GGAAATTGGGGTAGGAAGGA
Mouse Il12-F	TGGTTTGCCATCGTTTTGCTG
Mouse Il12-R	ACAGGTGAGGTTCACTGTTTCT

Supplementary Table S3. Antibodies and reagents in flow cytometry.

Antibodies and reagents	Source	Identifier
APC-Cy7 anti-mouse CD3	Biolegend	Cat# 100222; RRID: AB_2242784
APC anti-mouse CD3	Biolegend	Cat# 100236; RRID: AB_2561456
PerCP-Cy5.5 anti-mouse CD3	Biolegend	Cat# 100218; RRID: AB_1595492
FITC anti-mouse CD3	Biolegend	Cat# 100204; RRID: AB_312661
Brilliant Violet 605 anti-mouse CD4	Biolegend	Cat# 100451; RRID: AB_2564591
Brilliant Violet 711 anti-mouse CD4	Biolegend	Cat# 100447; RRID: AB_2564586
PE anti-mouse CD4	Biolegend	Cat# 100408; RRID: AB_312693
Brilliant Violet 605 anti-mouse CD8α	Biolegend	Cat# 100744; RRID: AB_2562609
Brilliant Violet 711 anti-mouse CD8α	Biolegend	Cat# 100759; RRID: AB_2563510
APC anti-mouse CD8α	Biolegend	Cat# 100712; RRID: AB_312751
Brilliant Violet 510 anti-mouse CD45.2	Biolegend	Cat# 109838; RRID: AB_2650900
FITC anti-mouse CD45.2	Biolegend	Cat# 109806; RRID: AB_313443
APC anti-mouse TCRβ	Biolegend	Cat# 109212; RRID: AB_313435
FITC anti-mouse TCRβ	Biolegend	Cat# 109206; RRID: AB_313429
PE-Cy7 anti-mouse TCRγ/δ	Biolegend	Cat# 118124; RRID: AB_11204423
FITC anti-mouse TCRγ/δ	Biolegend	Cat# 118106; RRID: AB_313830
APC anti-mouse CD11b	Biolegend	Cat# 101212; RRID: AB_312795
PE anti-mouse CD11b	Biolegend	Cat# 101208; RRID: AB_312791
Alexa Fluor 700 anti-mouse MHC-II	Biolegend	Cat# 107622; RRID: AB_493727
Brilliant Violet 421 anti-mouse MHC-II	Biolegend	Cat# 107632; RRID: AB_2650896
Brilliant Violet 785 anti-mouse Ly6C	Biolegend	Cat# 128041; RRID: AB_2565852
PE/Cyanine7 anti-mouse F4/80	Biolegend	Cat# 123114; RRID: AB_893478
Brilliant Violet 421 anti-mouse IFN-γ	Biolegend	Cat# 505830; RRID: AB_2563105
PE anti-mouse IFN-γ	Biolegend	Cat# 505808; RRID: AB_315402
Brilliant Violet 421 anti-mouse IL-17A	Biolegend	Cat# 512322; RRID: AB_11218604
PE anti-mouse IL-17A	Biolegend	Cat# 506904; RRID: AB_315464
Brilliant Violet 785 anti-mouse TNF-α	Biolegend	Cat# 506341; RRID: AB_2565951
APC anti-mouse IL-12/IL-23 p40	Biolegend	Cat# 505206; RRID: AB_315370
Alexa Fluor 488 anti-GFP antibody	Biolegend	Cat# 338008; RRID: AB_2563288
APC anti-mouse CD326 (Ep-CAM) antibody	Biolegend	Cat# 118214; RRID: AB_1134102
Purified anti-BrdU antibody	Biolegend	Cat# 364102; RRID: AB_2564498
BD Cytoperm™ Permeabilization Buffer Plus	BD Biosciences	Cat# 561651
BD Cytofix/Cytoperm	BD Biosciences	Cat# 554714
Fixation/Permeabilization Concentrate and Diluent	eBioscience	Cat# 00-5521-00
